# Paleoseismological evidence for segmentation of the Main Himalayan Thrust in the Darjeeling-Sikkim Himalaya

**DOI:** 10.1038/s41598-024-63539-1

**Published:** 2024-06-24

**Authors:** Atul Brice, R. Jayangondaperumal, Rao Singh Priyanka, Arjun Pandey, Rajeeb Lochan Mishra, Ishwar Singh, Madhusudan Sati, Pankaj Kumar, Sandipta Prasad Dash

**Affiliations:** 1 Wadia Institute of Himalayan Geology, Dehradun, Uttarakhand India; 2 Department of Geology, HNBGU, Srinagar, Uttarakhand India; 3 Department of Geology, University of Delhi, Delhi, India; 4 Inter-University Accelerator Centre, New Delhi, India

**Keywords:** Natural hazards, Solid Earth sciences

## Abstract

Whether the Main Himalayan Thrust can host a single surface-rupturing event in the Himalaya with a rupture length of > 700 km remains controversial. Previous paleoseismological studies in the Darjeeling-Sikkim Himalaya (DSH) suggested medieval surface-rupturing earthquakes, correlating them with the eleventh–thirteenth century events from Nepal and Bhutan and extending the coseismic rupture length > 700 km. Conversely, there is no rupture evidence of the 1714 Bhutan and 1934 Bihar–Nepal earthquakes in the DSH, resulting in a discrepancy in the rupture extent of the great earthquakes. Consequently, we conducted a paleoseismological investigation across a ~ 10 m-high fault scarp on the Himalayan Frontal Thrust at Chenga village, DSH, revealing a surface-faulting event during 1313–395 BCE. We suggest that the DSH is a 150 km-long independent segment bounded by a transverse ridge and fault and has a recurrence interval of ~ 949–1963 years, which is significantly larger than Nepal (~ 700–900 years) and Bhutan Himalaya (~ 339–761 years).

## Introduction

**Figure 1:**
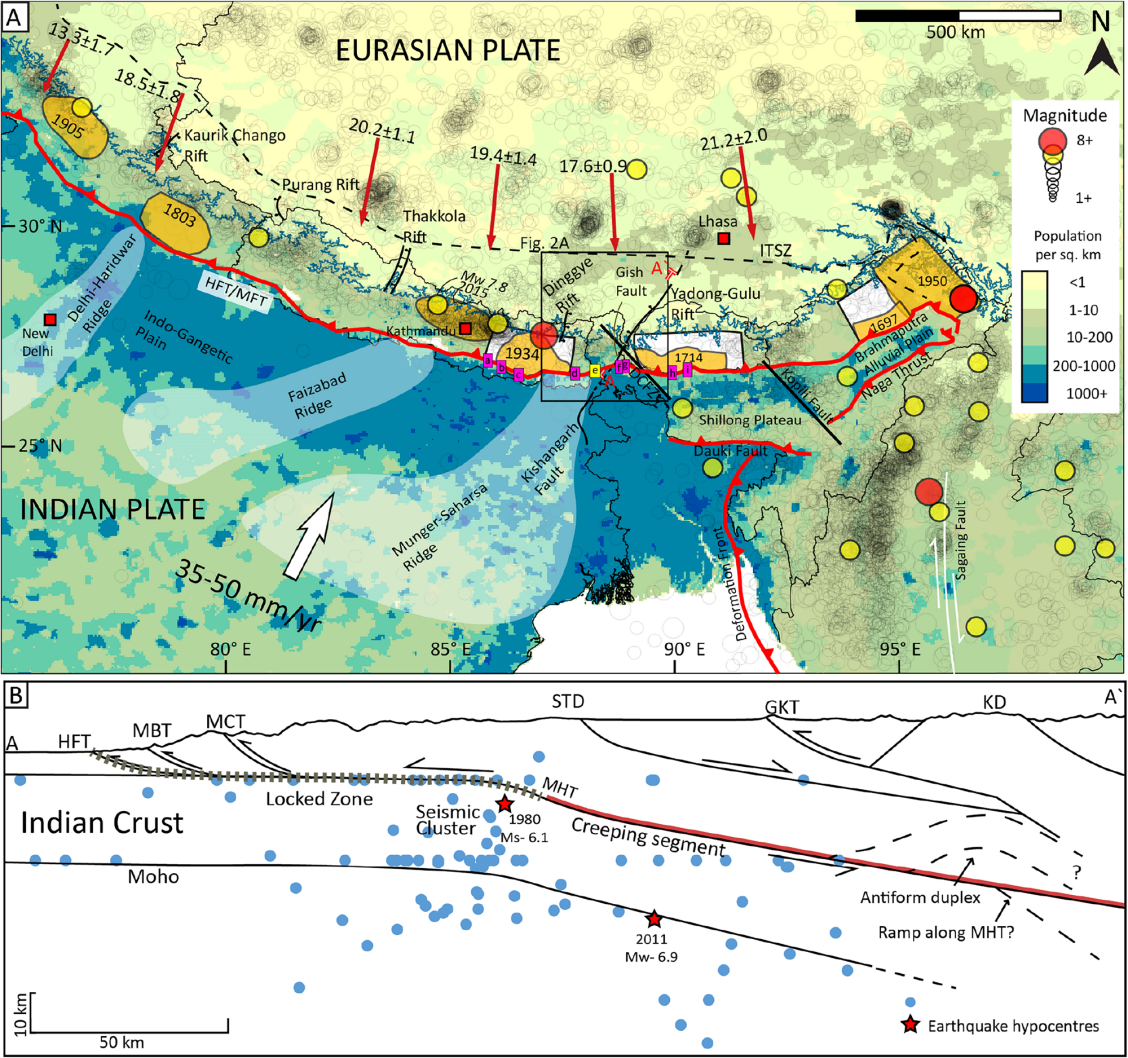
(**A**) A regional map of the Indian and the Eurasian Plates showing: black and white lines: major faults; meisoseismal zones and possible hypocentre location (translucent gold) of great earthquakes in the past millennia^20–24^; white polygons are the inferred rupture area of paleoearthquakes; white arrow: rate of motion of Indian plate and translucent white zones: sub-surface ridges^10,14^; blue line: ~ 3.5 km contour (locking-line); earthquake magnitudes (hollow circles- Mw < 7, solid-yellow circles: Mw7-7.9 and solid-red circles: Mw8+)^25^ and background with population density^26^. Red arrows: convergence rate^1^. Paleoseismic investigation sites: (a) Sir Khola^4^, (b) Charnath Khola^17^, (c) Khutti Khola^18^, (d) Damak^7^, (e) Chenga (this study), (f) Panijhora^5^, (g) Chalsa^3^, (h) Piping and (i) Sarpang^6,19^. (**B**) Cross-section (AAʹ), with major structures, are the locked and creeping segments of the Indian Plate^27,28^. Red star: hypocentre of 2011, Sikkim earthquake, blue dots: hypocentre of earthquakes (1965–2021)^25^. Abbreviations: DCFZ- Dhubri-Chungthang Fault Zone, GKT-Gyirong-Kangmar Thrust, HFT/MFT- Himalayan Frontal Thrust/Main Frontal Thrust, KD- Kangmar Dome, ITSZ-Indo-Tsangpo Suture Zone, MBT-Main Boundary Thrust, MCT-Main Central Thrust, STD-South Tibetan Detachment. The figures were created using Adobe Illustrator software, vCS-5, https://www.adobe.com/in/products/illustrator.

The rising Himalaya accommodates 13–21 mm/yr out of 35–50 mm/yr Indo-Eurasian convergence, where large-to-great earthquakes (*M*_w_ 7+) occurring at Main Himalayan Thrust (MHT) release a portion of accumulated elastic strain^1,2^ (Fig. 1A,B). Previous paleoseismological studies along the Himalayan Frontal Thrust (HFT) in India, Nepal and Bhutan revealed evidence of surface faulting mainly by two medieval earthquakes, i.e., 1100 CE and 1255 CE^3–7^. It raises the question of whether these medieval ruptures resulted from a single or separate contemporary event in different segments of the Himalaya. Assuming a single earthquake in Eastern Nepal, in Darjeeling-Sikkim Himalaya (DSH), and Bhutan, the rupture length would exceed 700 km^3,5,6^. While there is a record of *M*_w_ > 9 earthquakes along oceanic subduction zones^8^, it is still enigmatic whether such events are possible in the Himalaya^9^. Further, the orogenic segmentation hypothesis suggests that the Himalayan arc is segmented into smaller sections by transverse structures like ridges and faults^10–14^, contrary to its structural homogeneity^1,3,5,15^.

The DSH, which is ~ 150 km in length along its strike, lies between two transverse structures, i.e., the Munger-Saharsa Ridge in the west and the Dhubri-Chungthang Fault Zone (DCFZ^16^) in the east. It has no historical/recent record of a large-to-great (*M*_w_ > 7) earthquake (Fig. 1A,B), except for the debatable ~ 1100/1255 CE events^3,5^. Also, a considerable spatiotemporal gap exists in paleo-earthquake history in the DSH. However, it is enigmatic why great surface-rupturing events such as 1934 Bihar-Nepal^4,17,18^ and 1714 Bhutan^6,19^ are restricted to the periphery of the DSH bounded by the transverse structures. Towards this, we conducted a paleoseismological investigation at Chenga village in the DSH, India. Our results, combined with historical records and previous studies in the adjoining regions, suggest that the propagation of surface-rupturing earthquakes is controlled by transverse structures (Fig. 1A).

### Active tectonics in the DSH foothills and piedmont

**Figure 2:**
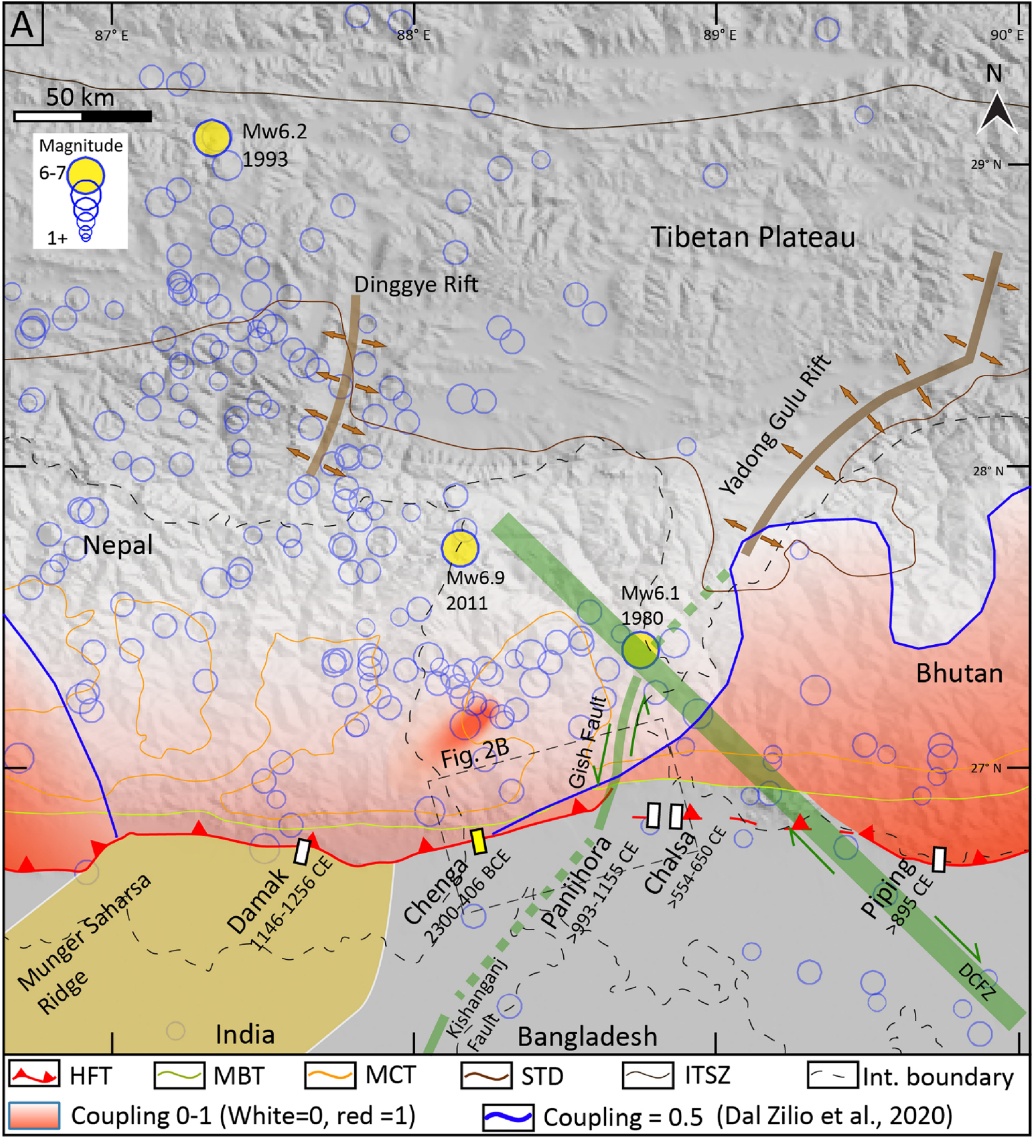

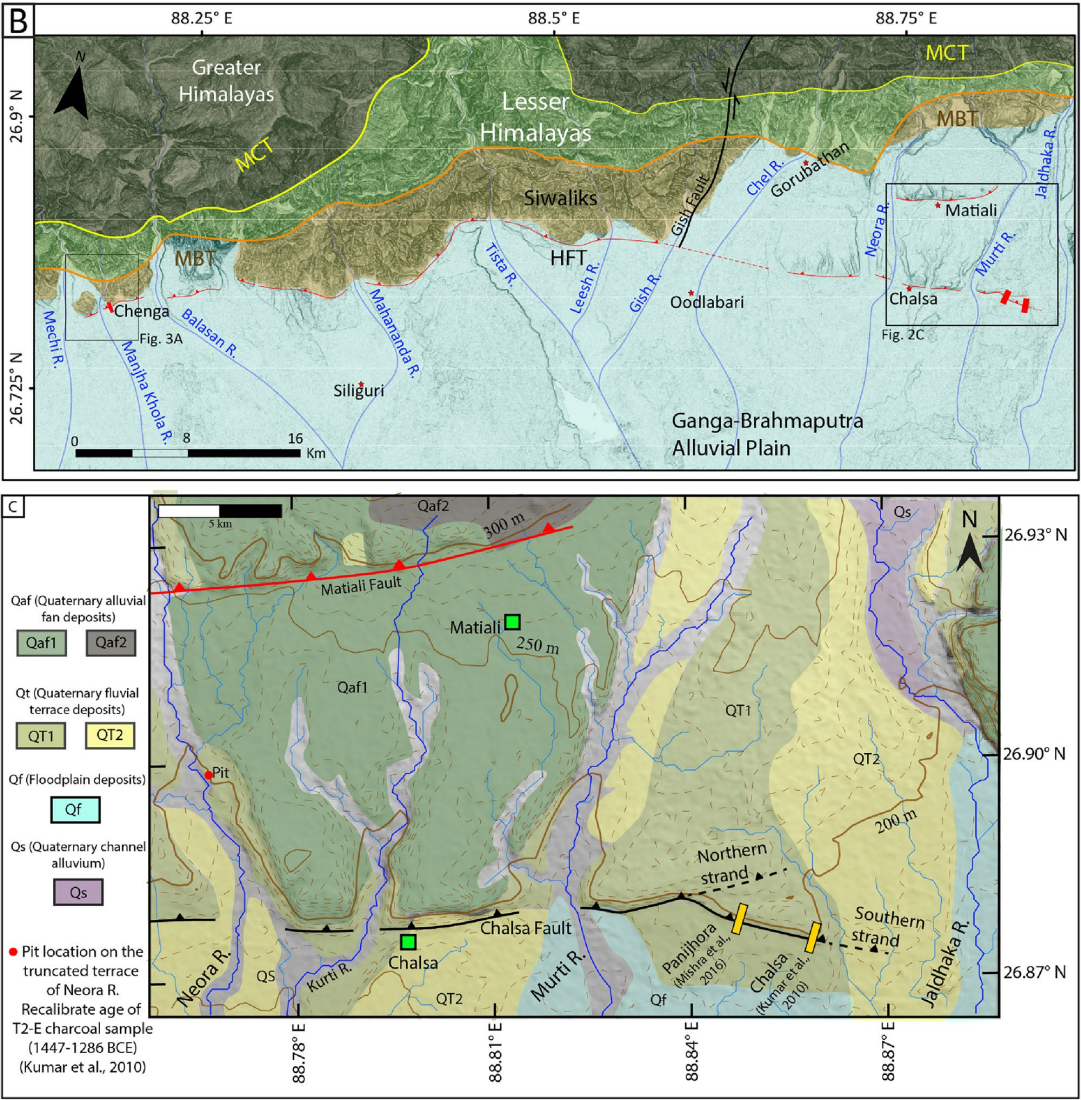
(**A**) Regional map with major faults and shadow zone between MSR and DCFZ on SRTM-90 m DEM (https://earthexplorer.usgs.gov/). Fading red: coupling on MHT with solid red being closer to 1^14^. Transparent blue circles are earthquake epicentres below 6, and solid yellow circles with 6–7 magnitude (https://earthquake.usgs.gov). Solid white rectangles: trench sites at Damak^7^, Panijhora^5^, Chalsa^3^ and Piping^19^, solid yellow rectangle: trench site of the present study at Chenga village. Abbreviations- DCFZ- Dhubri-Chungthang Fault Zone, HFT/MFT- Himalayan Frontal Thrust/Main Frontal Thrust, ITSZ- Indo-Tsangpo Suture Zone, LF- Lohargarh Forest, MBT-Main Boundary Thrust and MCT- Main Central Thrust. (**B**) The geology of DSH foothills and geomorphic features plotted on SRTM-30m DEM in the foothills are plotted along with the extrapolated HFT in the piedmont zone west of the Gish Fault^29,30^. (**C**) Topographic and geomorphic map of the Chalsa scarp on ALOS PALSAR DEM (12.5 m res) https://search.asf.alaska.edu/#/, indicating the location of the main fault trace that truncates the Neora River terrace and the location of trench investigations carried out by Kumar et al.^3^ and Mishra et al.^5^ across a smaller, younger fault scarp that splays towards south. The figures were created using Adobe Illustrator software, vCS-5, https://www.adobe.com/in/products/illustrator.

**Figure 3:**
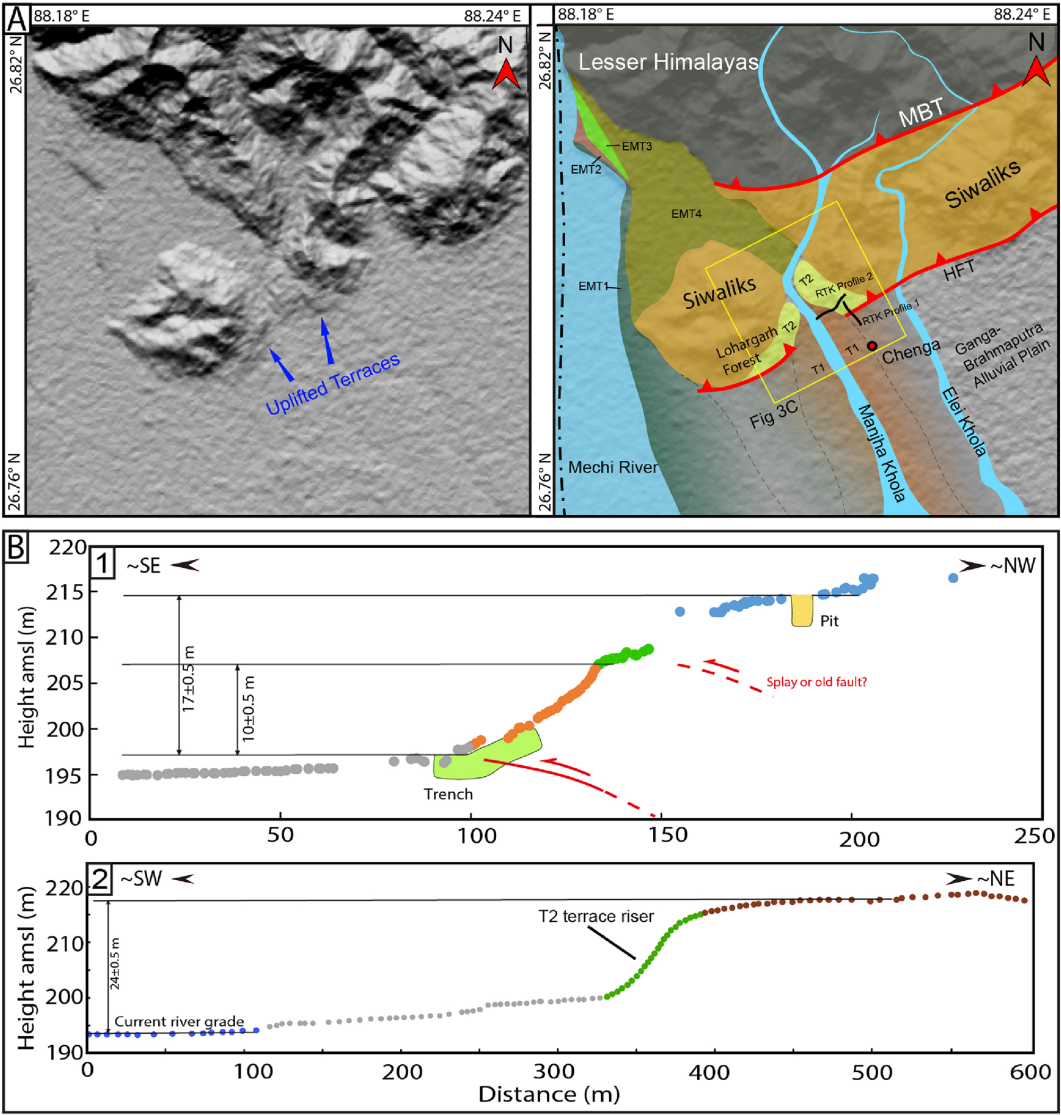

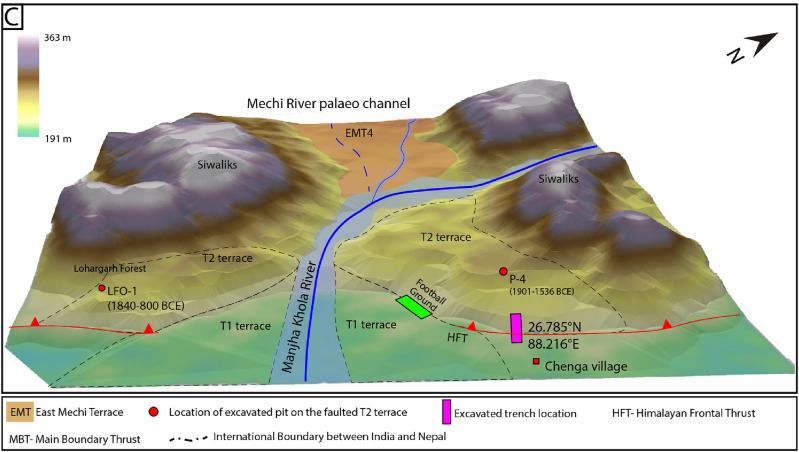
(**A**) Study area mapped on 12.5 m resolution ALOS-PALSAR DEM, https://search.asf.alaska.edu/#/. Red line: HFT (Himalayan Frontal Thrust). The first image is raw DEM with uplifted segments and topography; the second is the same region with mapped features. EMT- East Mechi Terrace, LF-Lohargarh Forest. (**B1**) 10 m and 17 m high scarp profiles extracted from the RTK-GPS survey. Yellow polygon: location of a pit excavated on the hanging wall. (**B2**) Profile across the terrace riser from current river grade to the surface of the truncated T2 terrace showing ~ 24 m elevation difference. (**C**) A geomorphic map of the study area was created using Triangulated Irregular Network method (1.5x) extracted from 12.5 m resolution ALOS-PALSAR DEM, using ArcGIS v10.5, https://www.arcgis.com/. Dashed lines mark two levels of terraces along the Manjha Khola River, where T2 terraces are truncated by HFT and T1 terraces are not cut by active fault. The figures were created using Adobe Illustrator software, vCS-5, https://www.adobe.com/in/products/illustrator.

Along the foothills of the DSH, the HFT is traced from the Mechi River in the west to the Gish River in the east (Fig. 2A,B). One peculiar change that occurs east of Chel River up to the DCFZ^16^ is the discontinuous presence of Siwaliks^29^ (Fig. 2B). The continuation of the HFT equivalent fault truncates the alluvial fans creating fault scarps in the piedmont zone of MBT where Siwaliks are not exposed, between Chel and Jaldhaka Rivers at Chalsa and Matialli villages^29^. Near the Eastern Indo-Nepal border in the western DSH, we identified fault scarps on the banks of Manjha Khola River at Lohargarh forest and Chenga village, based on field investigation aided with CARTOSAT-1 stereopair satellite data (2.5 m res) (Fig. 3A). We remapped the previously studied ~ EW striking Chalsa fault scarp^3,29^ using CARTOSAT (2.5 m res) and ALOS PALSAR (12.5 m res) DEMs and inferred that the easternmost part of the Chenga-Chalsa fault scarp splays towards the south with an NW–SE strike, forming a two-level 15–18 m high fault scarp (Supplementary Fig. S1). Kumar et al.^3^ and Mishra et al.^5^ carried out a paleoseismic trench investigation along this southern fault splay (discussed in detail in the subsequent sections). The Chenga-Chalsa fault scarps along the mountain front of DSH from west to east at Chenga and Chalsa villages have similar geometry with ~ 15–18 m high scarp^3,5^ (Fig. 2; Supplementary Fig. S1). However, towards ~ 55 km west of the present study site, i.e., Chenga, Wesnousky et al.^7^ identified a ~ 5.5 m high fault scarp along the HFT at Damak, Nepal, which formed during an earthquake event between 1146 and 1256 CE (Fig. 2A), and has a different geometry compared to Chenga-Chalsa fault scarps.

### Geomorphology of the study area

**Figure 4:**
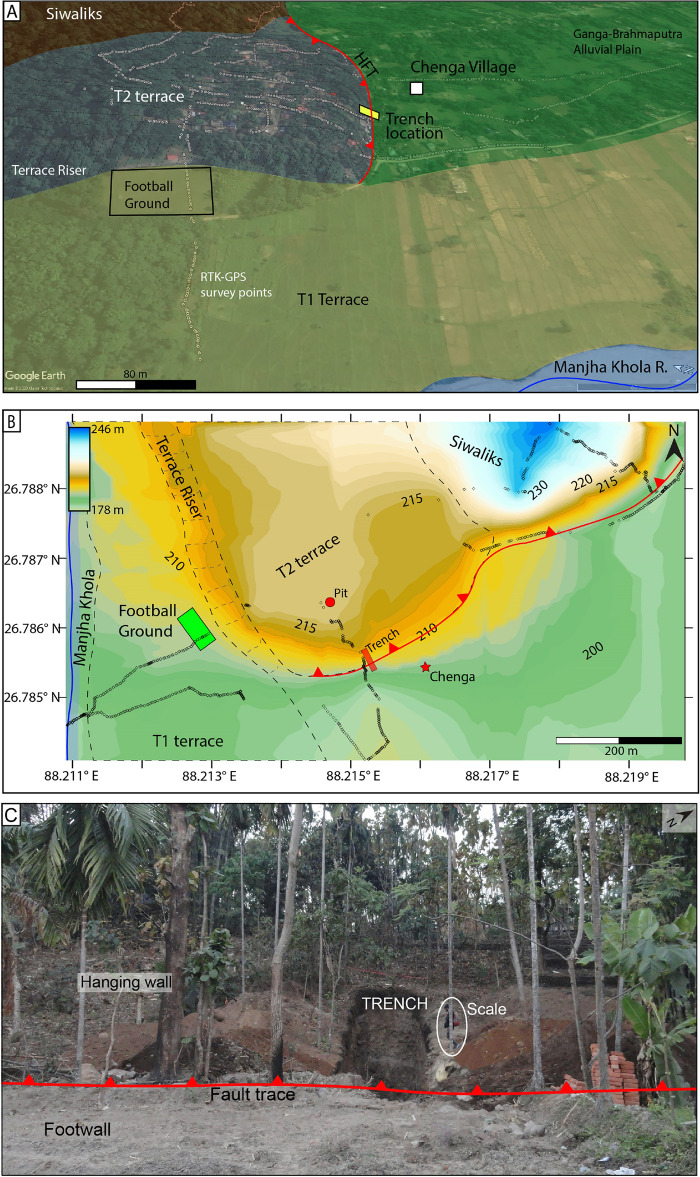
(**A**) Oblique view of the high resolution images available in Google Earth (v7.3.6.9750; https://earth.google.com/) of the fault scarp at Chenga village with RTK-GPS survey points and location of football ground at the base of the terrace riser. The top one is the raw image, and the bottom one is a labelled map of the terraces identified in the field. (**B**) Micro-topographic map of fault scarp at Chenga created from RTK-GPS survey data. The path created by black hollow squares is the data points collected from the RTK-GPS survey with ellipsoidal error < 1 m (gaps between the data points due to removal of high error points caused by the dense vegetated canopy). (**C**) Photograph of the fault scarp with trench excavated across the fault at the base of the scarp. The figures were created using Adobe Illustrator software, vCS-5, https://www.adobe.com/in/products/illustrator.

We conducted a paleoseismic investigation east of the eastern Indo-Nepal border (approximate course of Mechi River) along the HFT of DSH, West Bengal (Fig. 3A). Unpaired fluvial terraces are developed on the eastern bank of the Mechi River north of the HFT (Fig. 3A). The terraces are named EMT-1 to EMT-4 (East Mechi Terrace) based on their height, in the ascending order from current river grade. The EMT4 is a paleo-course of the Mechi River, which earlier merged with the Manjha Khola River between the Chenga village and the Lohargarh Forest (Fig. 3A,C). The EMT-2 to 4 lie north of the HFT, while EMT-1 extends along the eastern bank of Mechi River, and none of these terraces are cut by active faults. In the study area, two levels of paired terraces (T1 and T2) are preserved on either bank of the Manjha Khola, a tributary of the Mechi River (Fig. 3A,C). Only the T2 terrace is cut by the NE-SW striking HFT, and forms ~ 10 m and ~ 17 m-high stepped fault scarps (Fig. 3B). A trench (20 m long, 4 m wide and 5 m deep) was excavated across the ~ 10 m high fault scarp (Figs. 3, 4).

### Trench description

**Figure 5:**
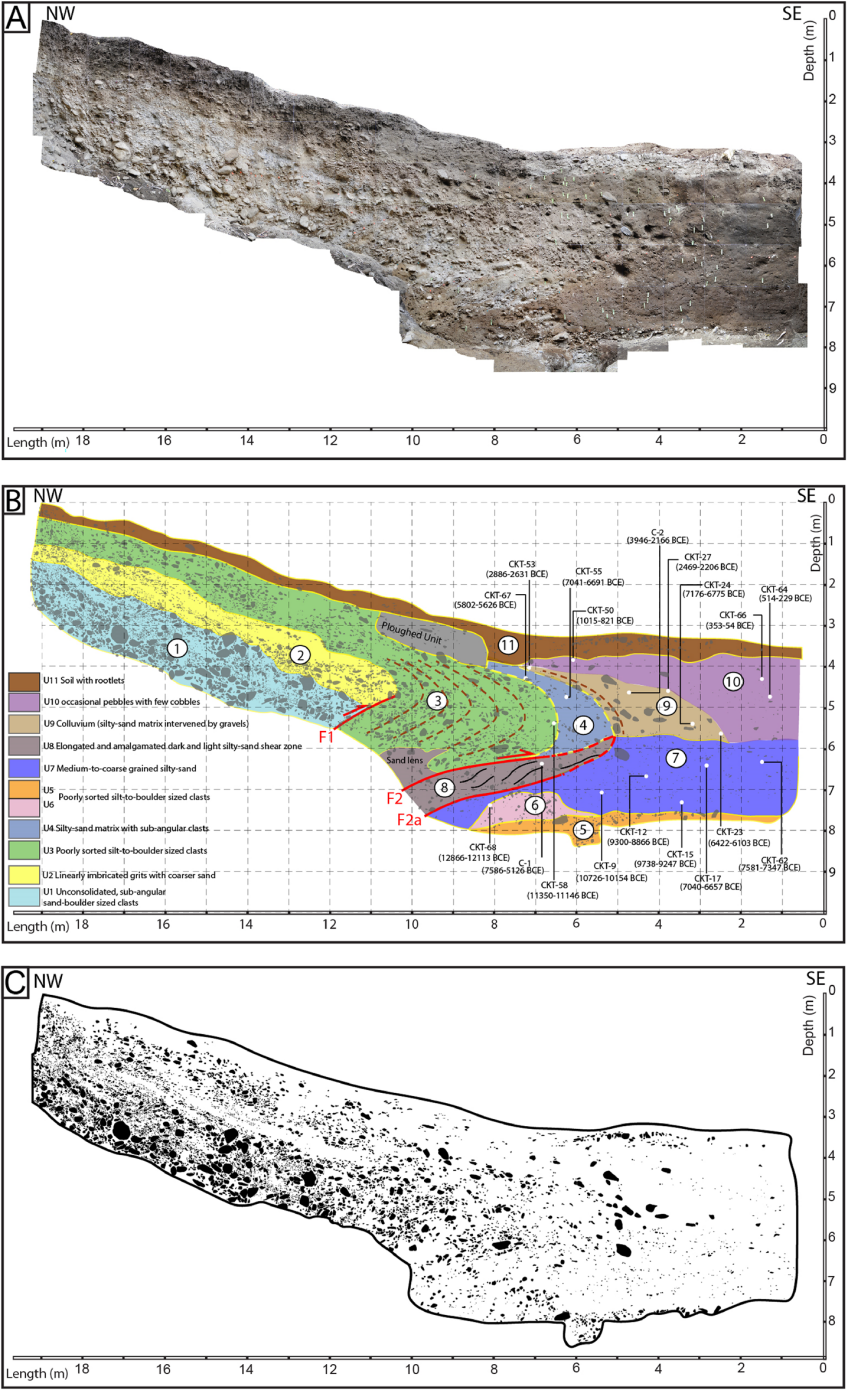
(**A**) Photomosaic of the trench excavated at the Chenga site mosaicked using Adobe Photoshop, vCS, https://www.adobe.com/in/products/photoshop.html. (**B**) Graphic representation of trench with unit description. Red lines: fault splays. (**C**) Illustrative log displaying the fabric of trench exposures. The figures were created using Adobe Illustrator software, vCS-5, https://www.adobe.com/in/products/illustrator.

Four units, namely unit 1–4, having a slope towards the south were identified in the hanging wall, which is deformed and displaced by three fault strands, i.e., F1, F2, and F2a. The fault strands F2 and F2a envelop a tapered shear zone towards its up-dip (Fig. 5). A minimum ~ 5.5 m coseismic slip was estimated along these fault strands. The lowermost unit-1 comprises ill-sorted, light brown coloured sub-angular boulders of size up to 40 cm in diameter intermingled with finer gravel and has a chaotic arrangement of clasts and matrix. Unit 2 comprises linearly imbricated grits with coarse sand, and a linear arrangement can be observed alternately in grits and coarse sands. It contains a silt-sized matrix and gravels up to 15 cm in diameter. It is partially oxidised and is marked by reddish-brown boulders and sand, indicating sub-aerial weathering. The units 1 and 2 terminate along the north dipping F1 fault. The matrix-supported gravel unit 3 shows folding along the F1 fault. In unit 3, planar fabric developed in the sediments tends to follow the antiformal fold geometry with hinge-thickening. Unit 4 comprises a light brown silty-sand matrix-supported gravel unit with relatively fewer clasts than unit 3, distinguished by a poorly defined boundary, which follows the fold geometry forming a nose near the hinge. At the base of the footwall, unit 5 is present, which is dominated by gravels of pebble-cobble size. It is a footwall equivalent of unit 3. It is overlain by a hump-like unit 6 just below the fault splay F2a with relatively fewer clasts varying in size from 2 to 20 cm, embedded within the brown medium-coarse sand matrix. Based on the shape of unit 6 and its resemblance to underlying unit 5 and being adjacent to fault splay F2a, it might be a dragged part of unit 5 due to fault movement. Unit 7 is thick dark brown, matrix-supported medium to coarse-grained silty-sand with sparsely distributed clasts of 1–5 cm large in diameter and overlays units 5 and 6. It has a texture similar to unit 4 but is dark in color. Unit 8 is the shear zone bounded between fault splays F2 and F2a. Ill-sorted, matrix-supported cobbles characterise this shear zone within the dark brown-light grey silty-sand matrix and display fault parallel fabric (marked as black wavy lines; Fig. 5B). Based on the texture, colour, and intermingling of brown sediments (unit-7) in grey gravel-dominant sediments (unit-3, 4), unit 8 is a possible result of the amalgamation and shearing of units 3 and 4 and 7. Overlying unit 7 is unit 9, composed of colluvium. It has dark brown coloured silty-sand. These gravels follow the curvature of the fold, indicating a deposition at the nose of the fold between 5 to 9 m horizontal scale marks. The matrix of this unit has a similar texture to units 4 and 7 and is likely a result of the erosion of units 3, 4, and 7. The colluvium unit is blanketed by unit 10, which is blackish-brown silty-sand with a diffused boundary. It has a rare occurrence of gravels and a few cobble-sized clasts. The topmost unit 11 along the slope of the hanging wall is greyish black soil covered with numerous rootlets, and on the flat top of the footwall wall, it is light grey-coloured, gravel-dominant filled material for the pavement of the road. Just beneath the soil cover in the hanging wall is a tilted, light brown coloured tabular unit that steeply cuts the adjoining units 3, 4, and 11. This small patch results from ploughing for agricultural use.

### Interpretation of trench and pit data

A total of 11 litho-units in the trench exposure are classified based on their grain size, shape and size of clasts, colour, matrix, arrangement of clasts, and energy condition of deposition (Fig. 5). The rock fabric of unit-1 is partly oriented, while gravels in the units 5 and 6 are randomly oriented. The planar fabric in unit-2 developed during alternate deposition of finer and coarser sediments, and it is deformed due to thrusting, uplift and warping/bulldozing. Orientation of the planar fabric of the rudaceous deposits in unit-3 tends to follow the fold geometry and gets thicker along its hinge zone. Charcoal samples were obtained from the units and dated with ^14^C AMS radiocarbon method to constrain the faulting event in the trench (“Methods”, ^14^C dating). The uncalibrated radiocarbon ages obtained in the present study and the radiocarbon ages of earlier studies concerned with our interpretation have been calibrated using the Bayesian technique in OxCal v4.4.4^31^ (Supplementary Table S1). The CKT-58 and 68 samples yield a similar age range of 12,886–11,146 BCE for units 3, 5, and 6. These units are overlain by overbank deposits comprising units 4 and 7, which yielded an age range of 7040–5626 BCE (CKT 67 and 17). Unit-4 capping the unit-3 at the hinge zone are folded. Unit 8, representing a shear zone, was devoid of charcoal. Hence, a sediment sample (C-1) which was obtained for Optically Stimulated Luminescence (OSL) dating, yielded an age 7586–5126 BCE, equivalent to ~ 7000 BCE radiocarbon ages from the unit-7 (CKT-17 and 62). This implies that the unit-8 was part of the unit-7 and sheared by the faults F2 and F2a. The ages of four samples CKT-23, 24, 27, and 53 derived from the colluvium unit-9 range from 7176–2206 BCE. The older age of ~ 6000–7000 BCE of samples CKT- 23 and 24, and the large boulder-sized clasts indicate that the colluvium is indeed derived from units 3 and 4. But the samples CKT-27 and 53 give a relatively younger age ~ 2000–3000 BCE, indicating syn-depositional age of the colluvium wedge. Unit-9 comprises cobbles aligned at the nose of the fold, indicating its origin by erosion of older units 3 and 4. An OSL sample C-2 obtained from the colluvium constrains its depositional age to 3946–2166 BCE (OxCal v4.4.4; Supplementary Table S2). The age of unit-9 is based on the minimum age model^32^ to reduce errors in overestimating depositional age caused by incomplete bleaching of quartz grains. Out of three charcoal samples, CKT-50, 64, and 66, obtained from unit 10, the samples CKT-64 and 66 are in stratigraphic order. But the oldest sample, CKT-50, lies atop at a shallower depth, which suggests that it is reworked. The capping of unit-10 indicates a period of no deformation; hence, the earthquake occurred before 514–229 BCE (CKT-64).

### Bracketing the age of the earthquake event at Chenga

**Figure 6:**
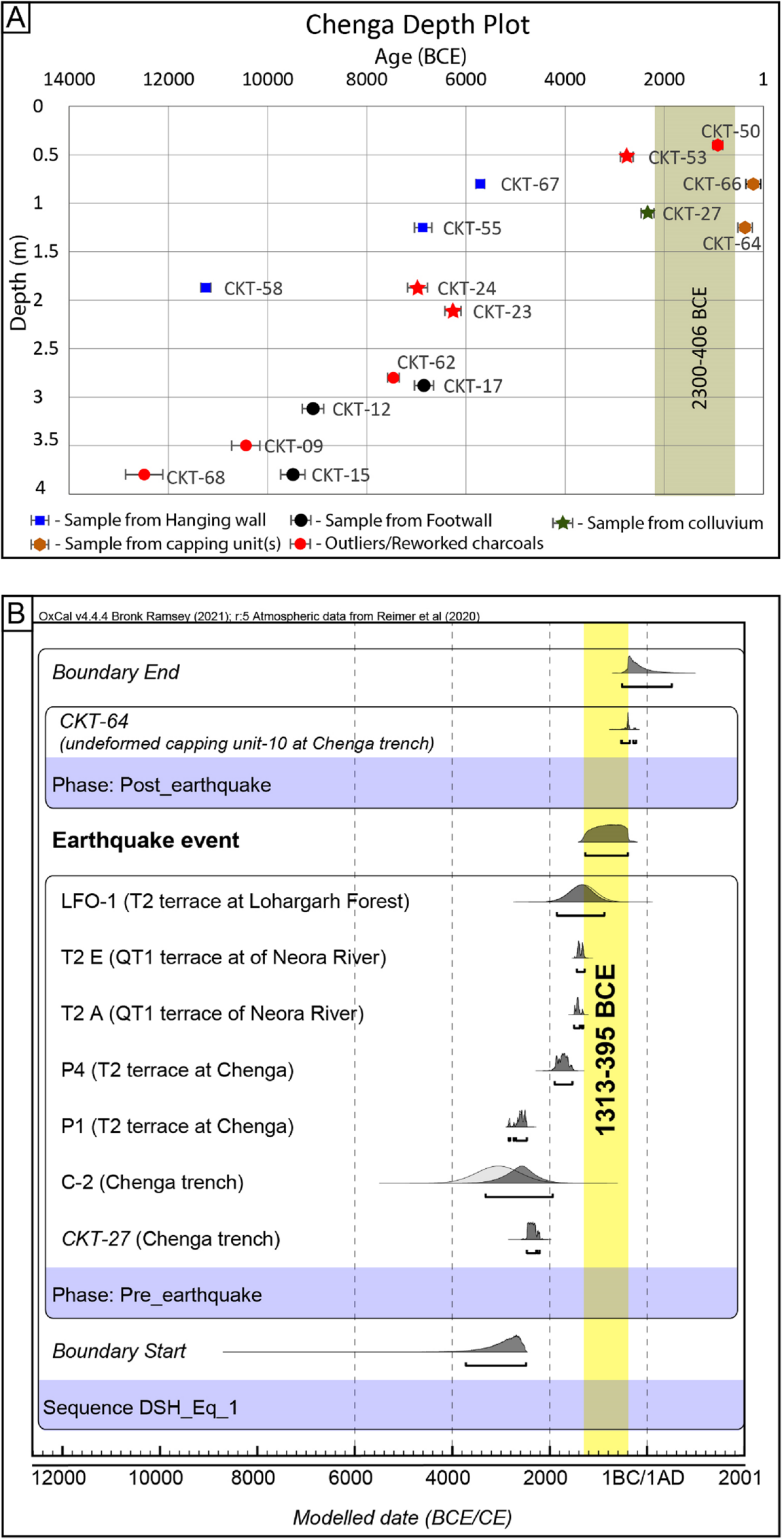
(**A**) Depth plot of radiocarbon ages of charcoal samples collected from the Chenga trench exposures. The hanging wall's blue dots override the footwall's black dots, indicating their emplacement due to thrusting. A broad greenish-brown stripe is the probable age range of earthquake occurrence. (**B**) A single rupture event hypothesis at all sites between 1313 and 395 BCE is marked in DSH based on the age correlation from trench investigation at Chenga, terrace abandonment of Manjha Khola River and Neora River^3^. The figures were created using OxCal v4.4.4^31^, https://c14.arch.ox.ac.uk/oxcal.html, and Adobe Illustrator software, vCS-5, https://www.adobe.com/in/products/illustrator.

The OSL age of colluvium unit-9 suggests that the earthquake occurred between 3946 and 2166 BCE, provided the sediments were well-bleached and the charcoals were emplaced after an earthquake. As the deformed units of the hanging wall are un-lithified sediments that would collapse to form colluvium soon with the formation of the fault scarp, the partial bleaching of the sediments will lead to overestimating the OSL ages. Therefore, we consider the youngest age 3946–2166 BCE for unit 9 as the lower bound of an earthquake event, which are synchronous with charcoal ages (CKT-27: 2469–2206 BCE). And the depositional age 514–229 BCE of the undeformed capping unit 10 as the upper bound. Thus, the earthquake event modelled using the OxCal program deduced that the recent displacement occurred between 2300 and 406 BCE (Fig. 6A).

A ~ 3 m-deep pit excavated on the faulted T2 terrace on the eastern bank of Manjha Khola (hanging wall of fault scarp at Chenga where trench was excavated) provided an abandonment age post-1901–1536 BCE (Figs. 3A,C, 4; Supplementary Fig. S2). Similarly, on the western bank of Manjha Khola, a ~ 1.25 m-deep pit was excavated on the surface of the T2 terrace at the Lohargarh Forest (Fig. 3C, Supplementary Fig. S2), which yielded an abandonment age 1840–800 BCE (OxCal 4.4.4; Supplementary Table S2). The abandonment age of the faulted T2 terrace on either banks of Manjha Khola River at Chenga (eastern bank) (i.e., post-1901–1536 BCE) is equivalent to that at the Lohargarh Forest (western bank) (i.e., post-1840–800 BCE), confirming a coeval co-seismic uplift. The age range of the event inferred from the trench coincides with that of the abandonment age of the T2 terrace of Manjha Khola, indicating that it was truncated between 2300 and 406 BCE (Fig. 6A,B).

**Figure 7:**
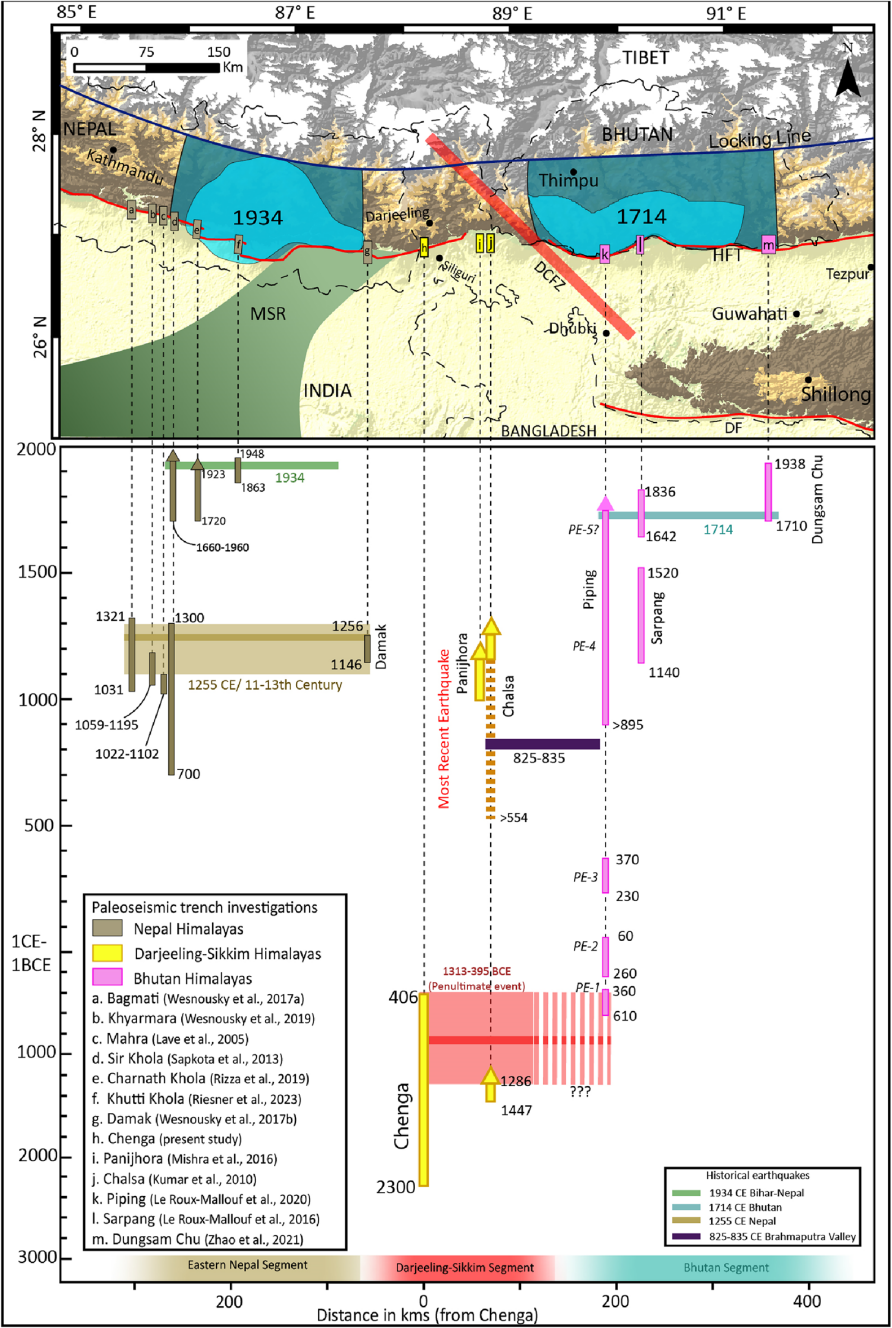
Space–time correlation with previous studies. The segment between the MSR and DCFZ demarcates the probable break in the Himalayan segment and the rupture extent of the 1255 CE, 1714 CE and 1934 CE events. meisoseismal zones and possible hypocentre location (translucent blue) of great earthquakes in the past millennia^20,21^; dark blue polygons are the inferred rupture area of paleoearthquakes; Locations of previous paleoseismic investigation sites in Nepal^4,7,17,18,28,37,38^, DSH^3,5^ and Bhutan^6,19,39^ region are allotted different colours that demarcate different segments. The vertical coloured bars are the age range of earthquake events inferred from numerous studies, and the bars with arrow head indicate no lower bound of the inferred earthquake, hence the event occurred post upper bounding age. The horizontal bars are the lateral extent of different paleoearthquake events. The dashed red bar of the penultimate event in DSH region implies a possible rupture beyond the DCFZ barrier. Three horizontal bars at the bottom of the figure with fuzzy (exact termination point unclear) ends represent the probable extent of different segments. The figure was created using Adobe Illustrator software, vCS-5, https://www.adobe.com/in/products/illustrator.

Previous work^3^ suggested the timing of an earthquake at the Chalsa fault scarp, eastern DSH, to be post 554–650 CE based on samples that lie beneath the fault trace in trench exposure. The authors also inferred an alternate speculation for a possible earthquake post-1162–1267 CE^3^ (OxCal v4.4.4: recalibrated in this study; Supplementary Fig. S3). The authors then correlated the latter inference in their trench with the ~ 1100 CE event reported earlier in Central Nepal^28^, rather than correlating with the events reported in near proximity^33^. Here, we confront the paleoseismological results with the historical and archeoseismological events described in the region for the comparison of various earthquake scenarios of post-554–650 CE and post-1162–1267 CE (Fig. 7). The first possible quake scenario may correspond to the 9th-century event (825–835 CE) that caused immense damage at Dhubri (~ 140 km SE of Chalsa trench site), Guwahati and Tezpur, covering a distance of ~ 280 km^33^. The second possible earthquake is related to the 1714 CE Bhutan earthquake, whose faulting evidence was reported at Piping, Bhutan, ~ 90 km east of Chalsa^19^ (Fig. 7). We suggest that either of the events mentioned above could have ruptured the Chalsa site, rather than the speculated ~ 1100/1255 CE events inferred in Nepal^4,7,28^. Also a pit on the surface of the truncated QT1 terrace of Neora River on the Chalsa fault scarp was excavated that lies ~ 9.5 km west of Chalsa trench site^3^. The abandonment age of the QT1 terrace of Neora River is post-1447–1286 BCE (OxCal v4.4.4), and it is comparable to the faulted T2 terrace of Manjha Khola River (Chenga: post-1901–1536 BCE and Lohargarh forest: post-1840–800 BCE). Interestingly, the height of these terraces from the current river grade is similar (i.e., ~ 25 m), indicating a coeval uplift phase for ~ 60 km along strike. This correlation implies an earthquake event occurred between 1313 and 395 BCE in the DSH.

The paleoseismic investigation at Panijhora^5^ is located ~ 1 km west of the Chalsa trench site on the same fault scarp, which was related to the historical 1255 CE earthquake^34^ that produced a surface rupture in Central Nepal^4^ (Fig. 2B). In the Panijhora trench, all the radiocarbon ages beneath the fault were considered reworked. The youngest charcoal sample (P-10: 993–1155) lies at a horizontal distance of 3.5 m from the up-dip of the fault trace at a depth of < 1.5 m in the center of massive unit-3 (Supplementary Fig. S4), inferring the event occurred post 993–1155 CE. This suggestion^5^ was later negated on claims of inadequate data presented towards the inference on the 1255 CE event in the region while supporting the initial hypothesis of ~ 1100 CE event^35^. Interestingly, the abandonment age of the QT1 terrace of Neora River, the inferred earthquake event in the Chenga trench and Manjakhola terraces in the present study do not correspond to the younger event inferred from Chalsa and Panijohora trench sites^3,5^, which need further interpretation. It could be due to degradation of the fault scarp by heavy rainfall (> 3000 mm/yr for the Darjeeling region^36^) or thrusting along the basal fault accompanied by a growth of footwall syncline or dragging syncline at the base of the fault. The latter proposition is evident in the radiocarbon ages of Chalsa trench exposures, where three deformed units are present with no capping unit (Supplementary Fig. S3). The trench exposures display a recumbent fold on the hanging wall (hanging wall anticline) and associated dragging syncline, thus doubling unit-3 in the footwall, between 28 and 38 m horizontal scale marks. The younger charcoals with an age range between 728 BCE and 648 CE (C-3, C-8 and C-E) lie at the center of unit-3, which is bounded by older radiocarbon ages above and below having an age range of 5539–408 BCE (C-1, C-3, C-9, C-D and C-F; Supplementary Fig. S3). This portrays the dragging syncline beneath the fault. Our reinterpretation and re-examination of the radiocarbon ages unit-3 in the Chalsa^3^ trench exposures support the assertion of footwall syncline. Hence, there is little to no colluvium in the Chalsa trench exposure due to deformation along the footwall syncline. In the Panijhora trench, the dragging syncline is not evident as compared to the Chalsa trench, which could be due to limited charcoal samples and smaller size of the trench exposure. The complex hidden structure may exist in the trench exposures. Therefore, careful examination of ages in complement with the tectonic geomorphological investigation in the adjoining trenched sites can better comprehend the paleo-earthquake history of the region. The Chalsa and Panijhora trench sites indicate that the southern strand of the Chalsa fault scarp reveals younger events, and the northern strand, the Chenga fault scarp, gives older BC events (Supplementary Fig. S1). This interpretation further suggests that steps like the Chenga-Chalsa fault scarp preserve more than one quake event.

### Correlating the rupture events of DSH

From the aforementioned inferences, we propose following scenario in the DSH. The penultimate event occurred between 1313 and 395 BCE preserved at the truncated T2 terraces of Manjha Khola (Chenga and Lohargarh Forest) and Neora River terraces^3^, while the Chalsa and Panijhora trench sites show younger events post 554–650 CE. From these observations, the recurrence interval in DSH is interpreted to be 949–1963 years.

**Figure 8:**
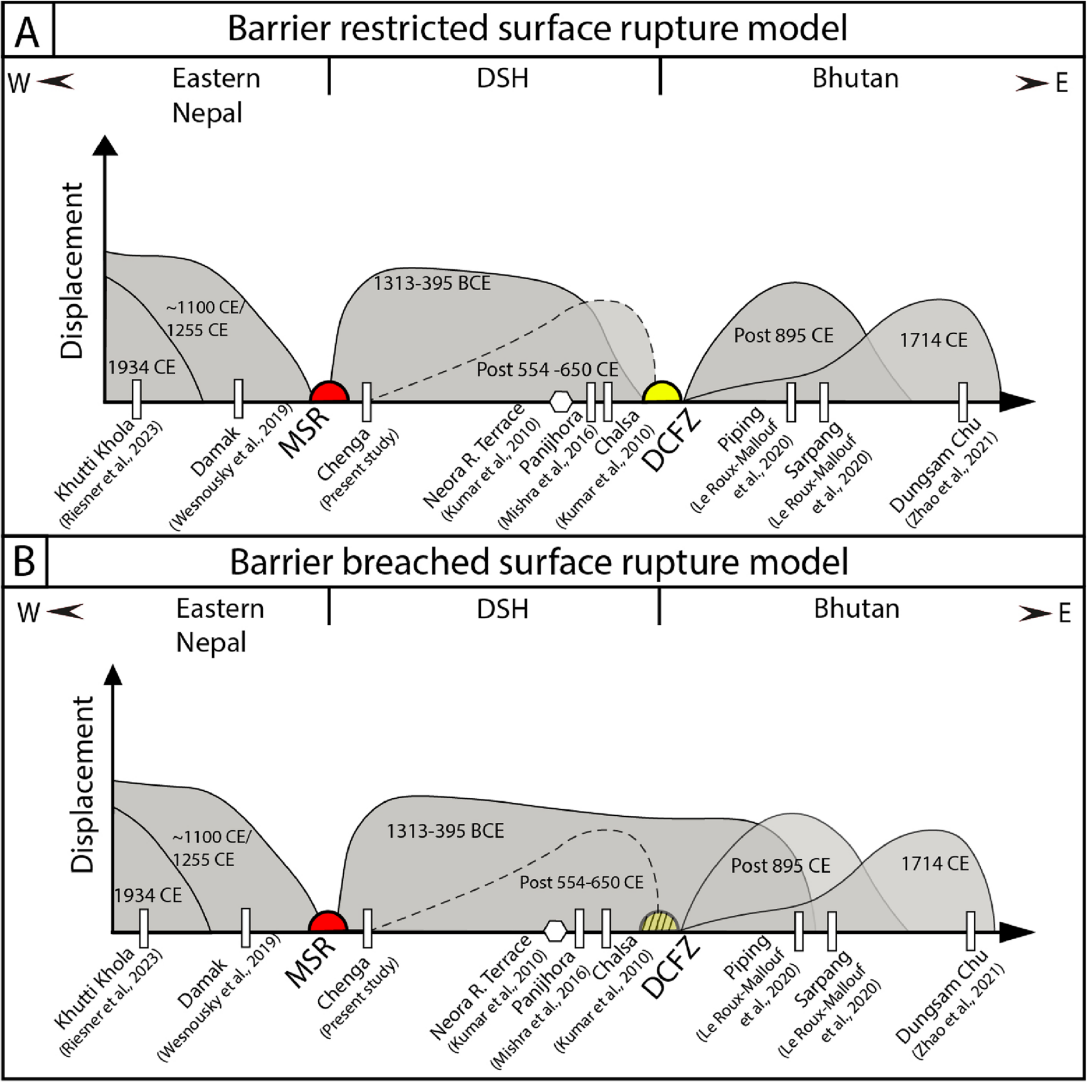
Schematic variable slip model of Eastern Nepal, DSH and Bhutan Himalaya showing fault displacement pattern where barriers (MSR—Munger-Saharsha Ridge and DCFZ—Dhubri-Chungthang fault zone) arrest the propagation of earthquakes (**A**) and the case where occasional propagation surpasses barrier (**B**).

Further ~ 90 km east of the Chalsa site at Piping, Bhutan, five events are inferred ranging from 610 to 360 BCE to post 895 CE, with a recurrence interval of 339–761 years from the paleoseismic exposure at a river-cut section in a 40 m-wide deformation zone^19^. They have proposed two possible earthquake events post-895 CE. The penultimate event occurred in the medieval period, and the most recent event was the 1714 CE Bhutan earthquake. The earthquake event(s) of Chalsa (post 554–650 CE) and Piping, Bhutan (post 895 CE) are comparable. The proximity of the Chalsa trench site from the Piping site (~ 90 km) and the age range of medieval earthquakes invite a possible correlation between the two locations. The earthquake event ‘E2’ at Chalsa could be related to the Piping events ‘PE4’ (9th–10th-century earthquake) reported at Piping (Figs. 7, 8). A similar correlation can be made between the earthquake event inferred in DSH (1313–395 BCE) and the ‘PE1’ event at Piping River cliff exposure (610–360 BCE) (Figs. 7, 8). The possible westward crossover-propagation of earthquake beyond the DCFZ can be supported by a ruptured segment boundary model, wherein rupture can occasionally propagate beyond it^40,41^ (Fig. 8B). However, there is no correlation between the earthquake event inferred at Chenga (1313–395 BCE) and ~ 55 km west at Damak, Nepal^7^ (1146–1256 CE), and these sites are separated by the eastern extent of the Munger-Saharsa Ridge, suggesting that it acts as a barrier terminating the rupture propagation of earthquakes (Figs. 7, 8).

### Segmentation and earthquake propagation in Eastern Nepal, DSH and Bhutan Himalaya

Based on radiocarbon ages, we interpret two events in the DSH along Chenga-Chalsa fault scarp during 1313–395 BCE and post 554–650 CE. However, in the eastern Nepal, the previously inferred events occurred during ~ 1100/1255 CE and 1934 CE, whereas post 895 CE and 1714 CE in Bhutan. Further indicating that each segment ruptures independently, and rupture propagation is limited due to the basement transverse structures, Munger-Saharsa Ridge and DCFZ to the west and east, respectively, favouring the MHT segmentation hypotheses (Figs. 7 and 8A). Numerous geophysical studies documenting orogenic segmentation in the Himalaya advocate for each segment to behave as an independent seismogenic entity bounded by transverse structures to arrest the rupture extent of great earthquakes^10–12,14^. The dominance of active seismicity along such transverse structures is evident from the instrumentally recorded major earthquakes in the DSH, for example, 1980 (*M*_w_ 6.2) and 2011 (*M*_w_ 6.9) earthquakes^42,43^ (Fig. 2A). Additionally, the along strike structural segmentation of the MHT will reduce the possibility of a giant megathrust earthquake (*M*_w_ > 9) in the Himalaya due to the smaller segments rupturing independently.

### Strain dissipation and prolonged recurrence in the DSH

The DSH differ from Himalayan regions to the east and the west^44,45^. The decollement here is narrower than elsewhere and coincides with the pole of rotation of the Brahamaputra block, east of which convergence rates on Himalayan faults slow as a result of the Brahmaputra's clockwise rotation relative to India^44^. The absence of a foredeep north of the Shillong plateau, east of the Dhubri fault is a third cogent reason for supposing that rupture may encounter different physical conditions and frictional properties along strike^44^. Few reasons that justify the prolonged recurrence of a great surface rupturing event at HFT of DSH are (a) lower inter-seismic coupling in DSH than in adjoining regions^9,13,14^, (b) strain transfer along the out of sequence thrusts and internal shortening within the Himalayan wedge^46–48^, and (c) contrasting geometry of the MHT at DSH in comparison with rest of the Himalayas^49,50^. We suggest that lower seismic coupling, strain dissipation within the Himalayan wedge and a ramp structure at the frontal MHT significantly prolongs the recurrence period of large to great earthquakes in the DSH.

Our study establishes three independent segments, i.e., Eastern Nepal, DSH in India and Bhutan, having different recurrence intervals of 700–900 years^51^, 949–1963 years (this study), and 339–761 years^19^, respectively (Figs. 7, 8). Compared to adjoining regions, the recurrence interval of great surface-rupturing events of DSH is more prolonged (approximately 1.5–2× than Nepal and 2–3× than Bhutan). Complex tectonics of DSH further invite rigorous paleoseismic research work at close spatial intervals for a better insight into assessing the seismic hazard.

## Methods

### Lab work and field survey

The Darjeeling-Sikkim region's geological and geomorphic maps (1:25,000 scale) were explored to set a base for the active tectonic investigation in the study region. This was further aided by CARTOSAT stereopair imageries (2.5 m resolution), which were then analysed using Socet software for 3-D visualisation of the possible fault scarps along the mountain front. The study region was surveyed using RTK-GPS to log high-precision (~ 1 cm accuracy) tectonic geomorphology. The tectonic scarps were surveyed on foot to log their morphometrics. Drone photographs were taken to check the lateral extension of the structural features (tectonic) (Supplementary Fig. S5).

### ^14^C dating

We collected 21 detrital charcoal samples from the excavated trench (16) and pit (5) across the fault scarp at Chenga village, Darjeeling. Nine charcoal samples from the trench were analysed at Poznań Radiocarbon Laboratory, Poznań, Poland, and seven charcoal samples from the trench and five from the pit were analysed at IUAC, New Delhi, India. The collected charcoal samples were first pre-treated physically. The clean charcoal samples were chemically pre-treated using standard Acid–Base-Acid pre-treatment. The samples were then frozen solid. After that, the frozen samples were freeze-dried in a vacuum for 6 h. The dried samples were packed in small tin boats and combusted in an Elemental Analyzer. The Automated Graphitization Equipment then produced graphite by reducing the CO_2_ from sample combustion with H_2_ on the iron catalyst. The graphite formed was then transferred to a cathode and pressed with a force of 150psi. Finally, the samples were analysed using Accelerator Mass Spectrometry at IUAC, New Delhi, India. The results are given in Supplementary Table S1.

### Sediment sampling and analysis

We collected sediment samples from two units of the excavated exposures of a trench by hammering ~ 25 cm long and ~ 5 cm in diameter steel tubes without exposing the sediments to sunlight. These samples were processed and analysed at the TL/OSL lab at Wadia Institute of Himalayan Geology (WIHG), Dehradun, India. The analysis was carried out in controlled darkroom conditions. Carefully top and bottom 2–3 cm sediment was kept aside and analysed to determine U, Th and K concentration in the samples at XRF lab, WIHG, Dehradun. The remaining sample(s) were dried to measure approximate water content. The dried samples were then sieved (75–125 microns) and treated with 1N HCl and conc. H_2_O_2_ to remove carbonates and organic matter. Quartz grains were then separated using heavy liquid (sodium poly tungstate). The collected quartz samples were treated with 40% HF for 80 min and 20 min in conc. HCl with intermittent stirring to etch outer ~ 20 microns and dissolve feldspar contamination. The purity of quartz was done using the Infra-Red Stimulated Luminescence test. The dose calculations were done using the Riso TL/OSL-12 system. The paleodose concentrations were measured using Single Aliquot Regeneration (SAR) protocol^52^. From the acquired equivalent dose and U, Th and K concentrations in the sample, the depositional age of the sediments was estimated using Luminescence Dose and Age Calculator (Excel-based program)^32^ (Supplementary Fig. S6, Table S2).

### Supplementary Information

Supplementary Information.

## Data Availability

All data generated or analysed during this study are included in this published article [and its supplementary information file].
