# Supplementary material for: Paleoseismological evidence for segmentation of the Main Himalayan Thrust in the Darjeeling-Sikkim Himalaya
Source: Sci Rep. 2024 Jun 24;14:14537. doi: 10.1038/s41598-024-63539-1 (PMC11196676; doi:10.1038/s41598-024-63539-1)
Supplement: Supplementary file 1 — Supplementary Information. [file 41598_2024_63539_MOESM1_ESM.docx]

**Paleoseismological evidence for segmentation of the Main Himalayan Thrust in the Darjeeling-Sikkim Himalaya**

Atul Brice^1,2^, R. Jayangondaperumal^1^*, Rao Singh Priyanka^3^, Arjun Pandey^1^, Rajeeb Lochan Mishra^1^, Ishwar Singh^1^, Madhusudan Sati^2^, Pankaj Kumar^4^, and Sandipta Prasad Dash^1^

*^1^Wadia Institute of Himalayan Geology, Dehradun, Uttarakhand, India*

*^2^Department of Geology, HNBGU, Srinagar, Uttarakhand, India*

*^3^Department of Geology, University of Delhi, Delhi, India*

*^4^Inter-University Accelerator Centre, New Delhi, India*

**Correspondence to: R. Jayangondaperumal (ramperu.jayan@gmail.com)*


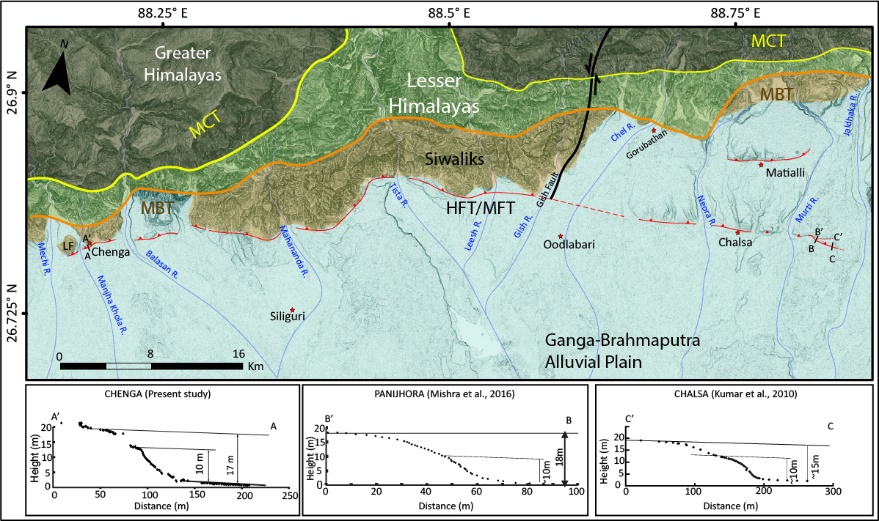
**Figure S1.** Major geomorphic features mapped on SRTM-30m DEM (<https://earthexplorer.usgs.gov/>) in the foothills are plotted along with the extrapolated HFT in the piedmont zone west of the Gish Fault^1,2^. The scarp profile comparison across the fault scarps at Chenga (A-A’), Panijhora (B-B’) and Chalsa (C-C’) inferred from the RTK-DGPS survey^1,3^.


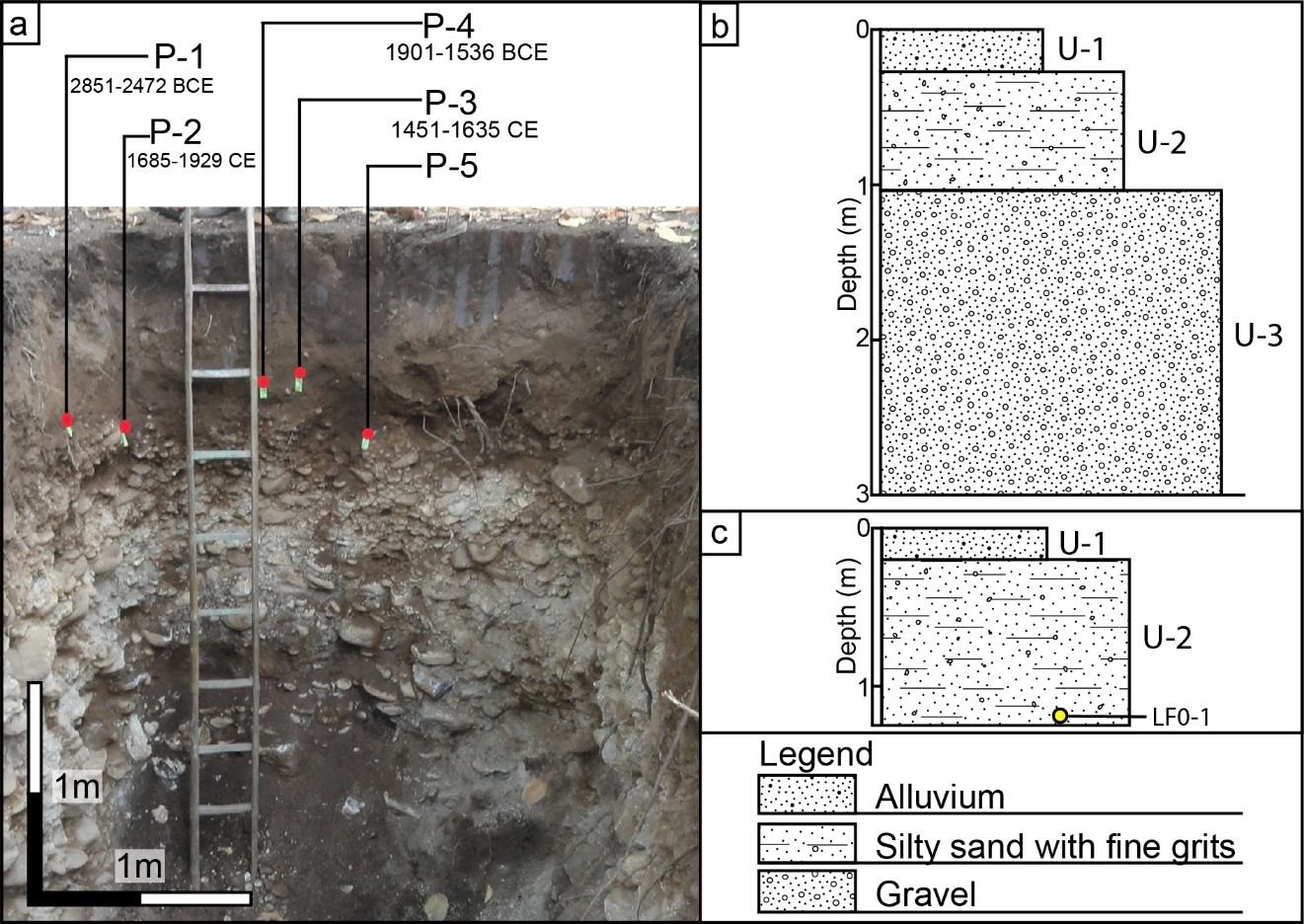
Figure S2. (a) Pit photograph at the surface of the T2 terrace at Chenga, with the location of charcoal samples collected to infer the abandonment age of the T2 terrace. (b) The log of this pit shows ~20cm thick alluvium cover at the top composed of dark brown soil with numerous rootlets. ~85cm thick brown silty-sand unit follows with fine grits in middle and ~2m thick gravel unit at the base with unknown depth with alternating brown and grey clasts varying in size from 2cm to >50cm. (c) Log of the pit excavated at T2 terrace at Lohargarh forest. The top unit is ~20 cm thick alluvium and is composed of bioturbated medium-grained soil with numerous roots. The lower unit is at least 1m thick with medium to coarse silty sand with finer gritty material. The yellow circle is the location of sediment sample LFO-1 collected to carry out OSL dating to infer the abandonment age of the T2 terrace.


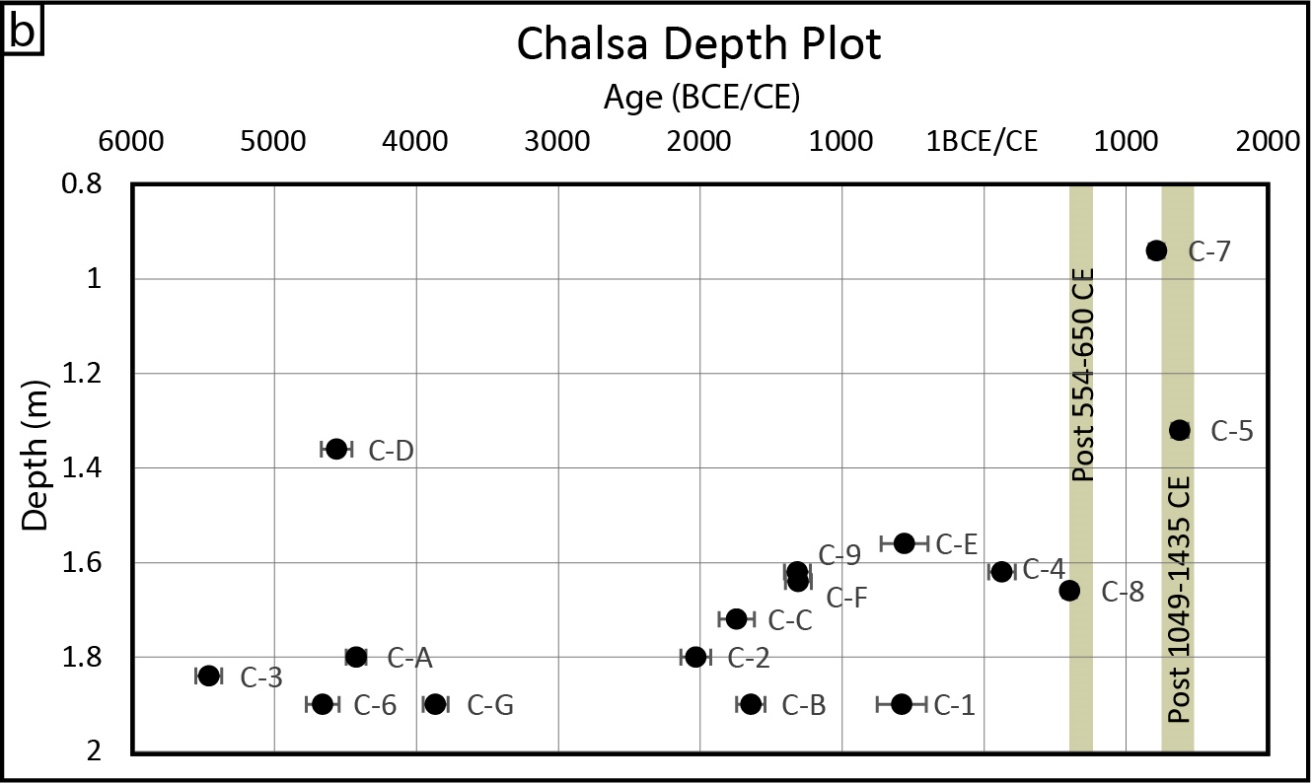

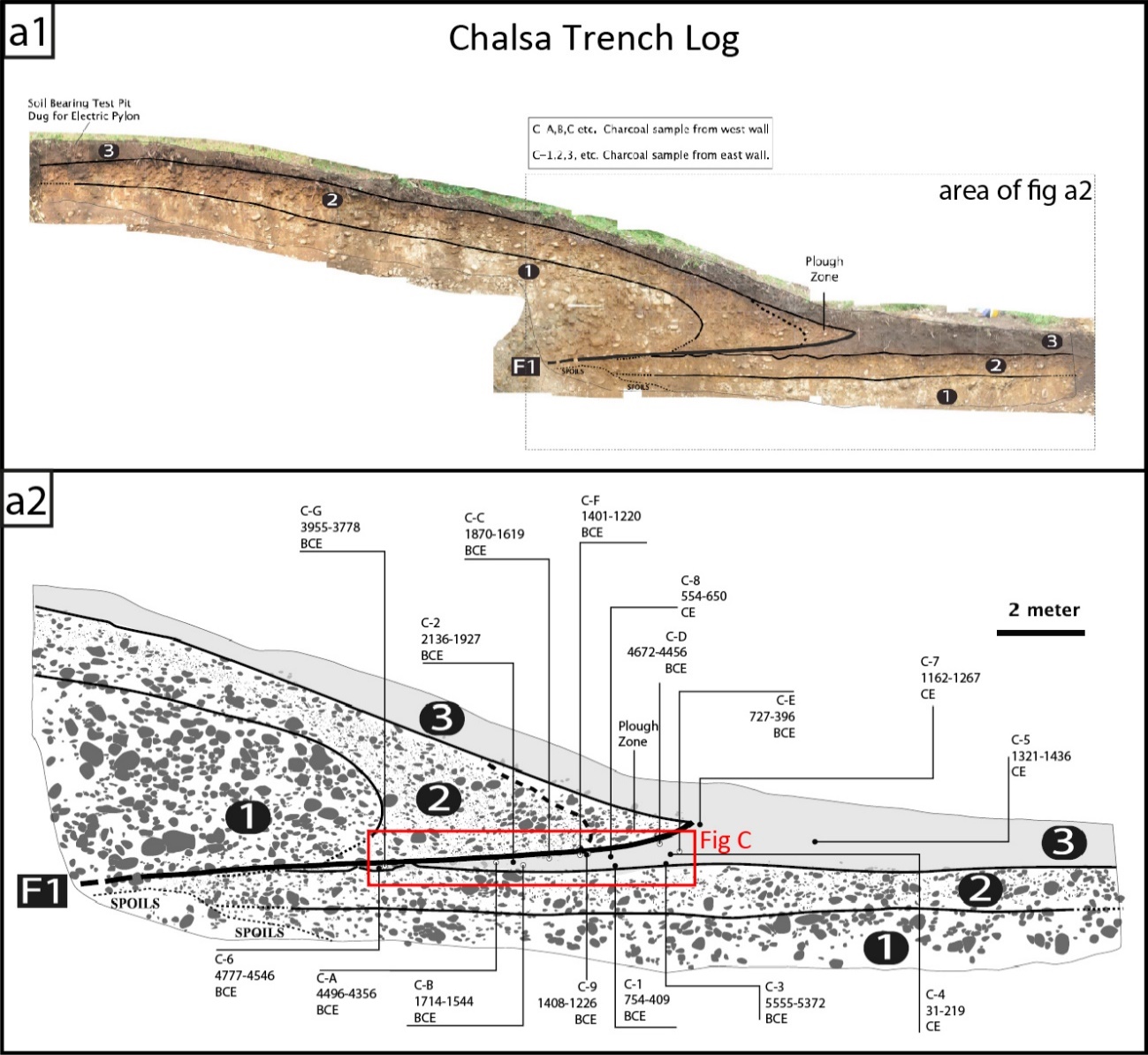


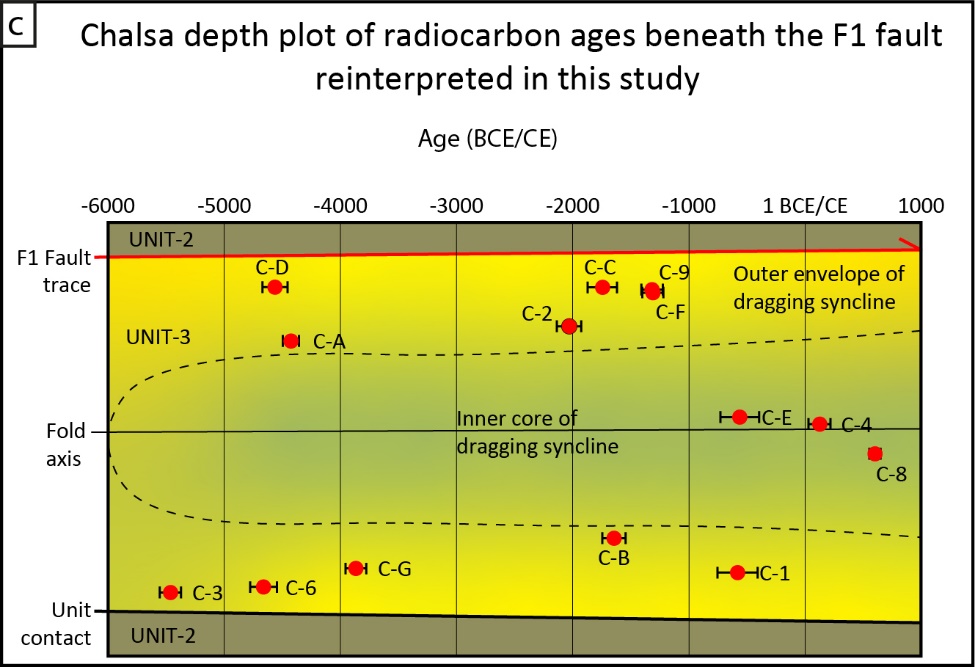
Figure S3. (a) Trench log of Chalsa site as published in Figures 5 and 6 of Kumar et al.^1^. The radiocarbon dates of the samples have been re-calibrated using OxCal 4.4.4^4^. (b) Depth plot of recalibrated radiocarbon dates. Two possible earthquake events are inferred: first, post 554-650 CE, based on the location of sample C-8 beneath the fault scarp and second, post 1049-1435 CE if the earthquake event occurred subsequent to the deposition of unit-3. (c) Schematic depth plot of the radiocarbon charcoal ages collected beneath the fault scarp. The relative position of the charcoal samples are plotted that were collected from the trench exposure between the fault contact and the contact of units 2 and 3. These ages imply the presence of a footwall syncline due to the dragging of unit 3 along the fault.


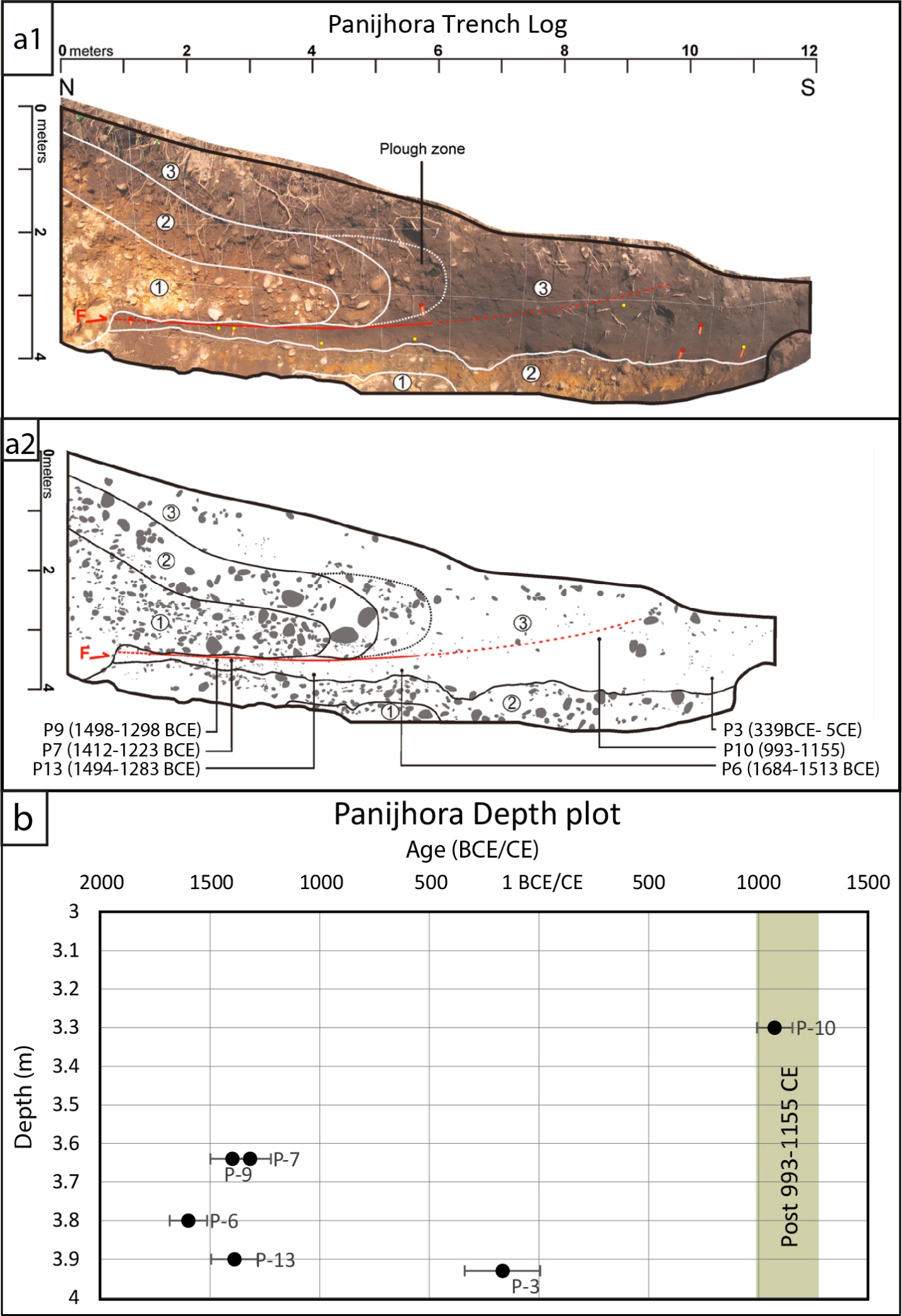


Figure S4. (A) Trench log of Panijhora site as published in Figure 3 a, b of Mishra et al.^3^. The radiocarbon dates of the samples have been re-calibrated using OxCal 4.4.4^4^. (B) Depth plot of recalibrated radiocarbon dates. The earthquake event occurred post 993-1155 CE subsequent to the deposition of unit-3.


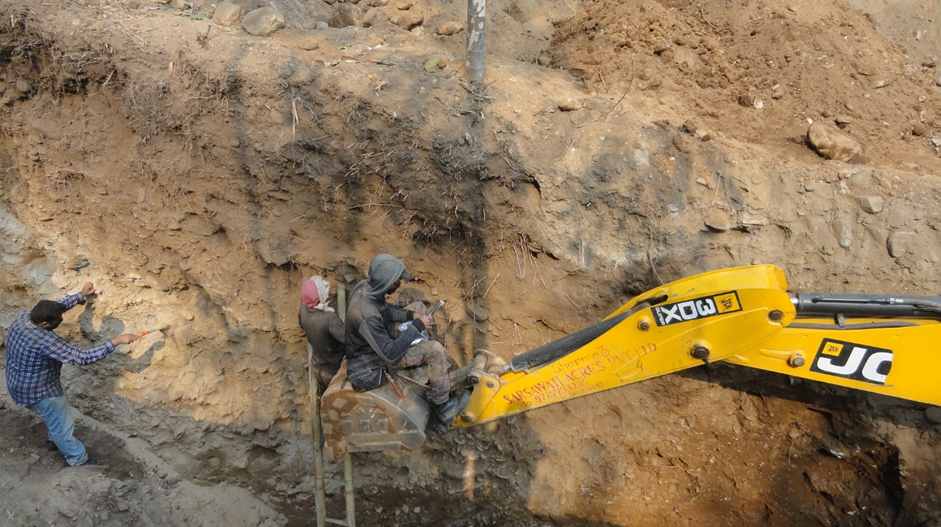

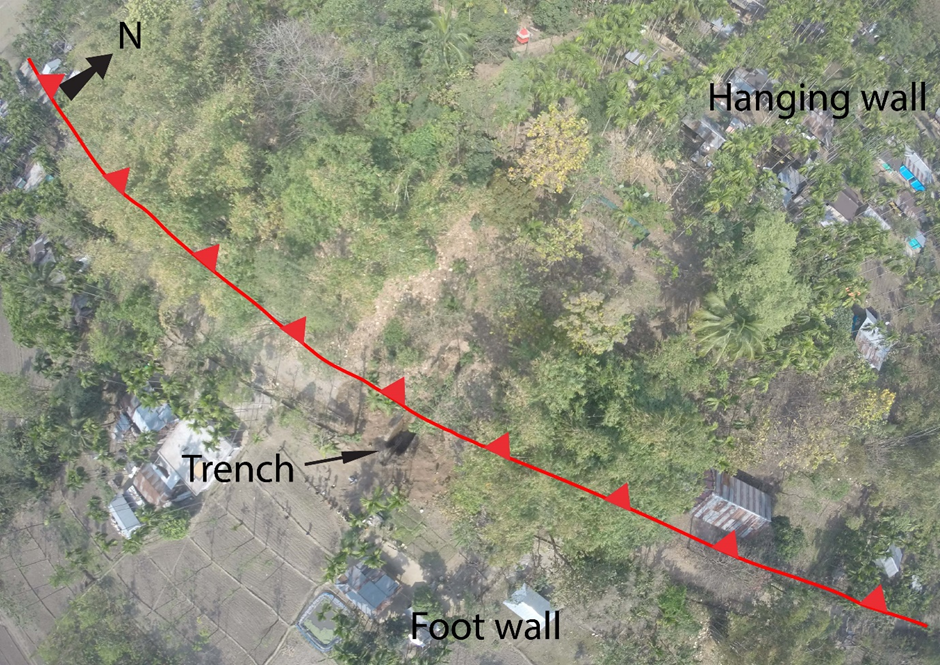
Figure. S5. The top image is the bird’s eye view of the fault scarp with an excavated trench location at the base of the scarp. The photograph was taken using the Ricoh GR digital camera, which was mounted on DJI Phantom 1 unmanned aerial vehicle. The bottom left portion shows farmed flatland (footwall), and the top right portion is the uplifted fault scarp (forest and village area) (hanging wall). The bottom image is the view from the ground surface of the trench carrying out scraping of the walls and collecting charcoal samples.


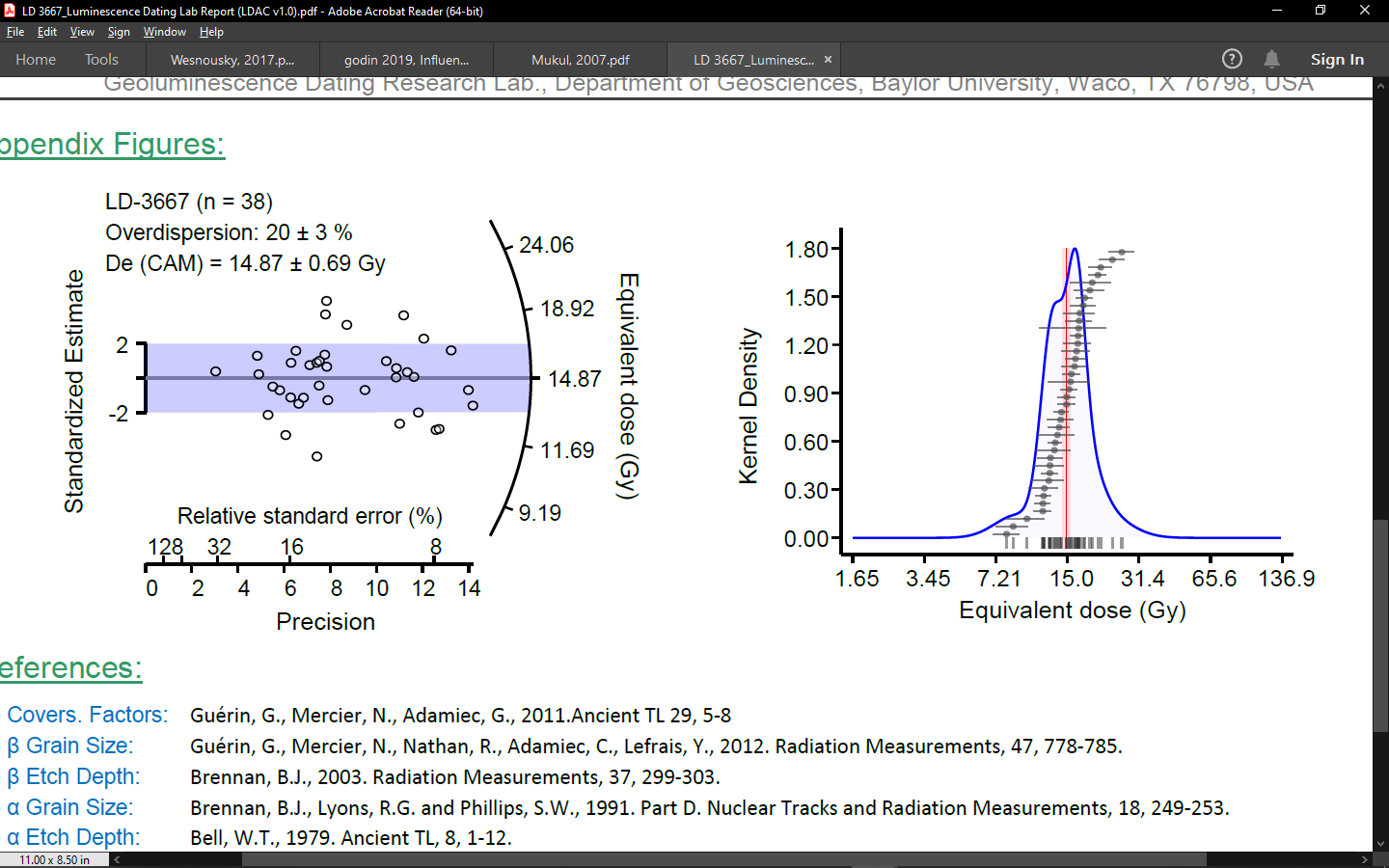


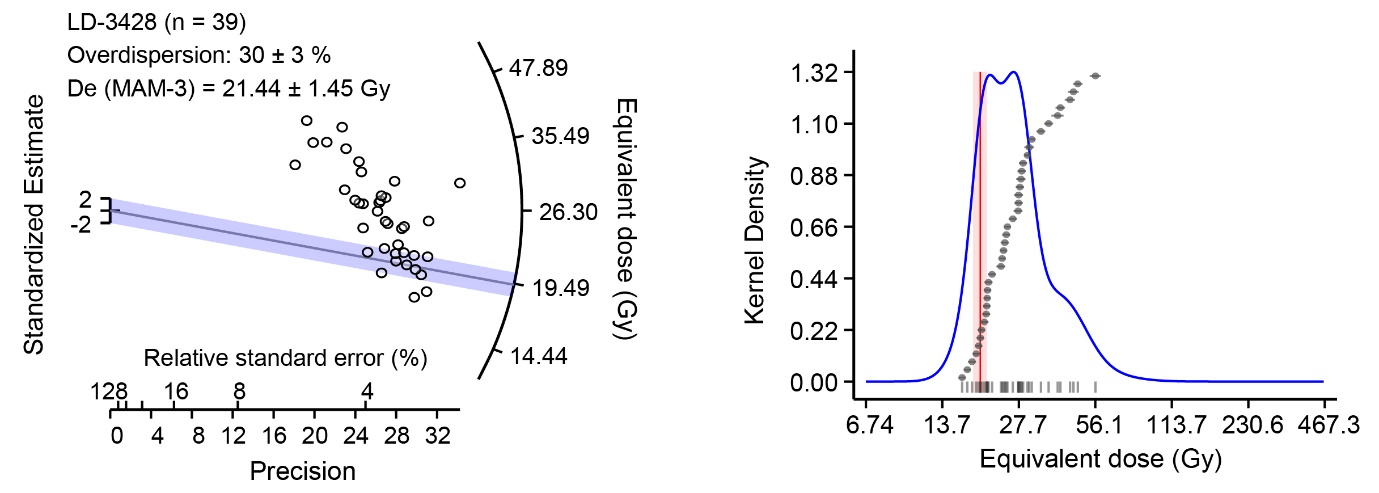

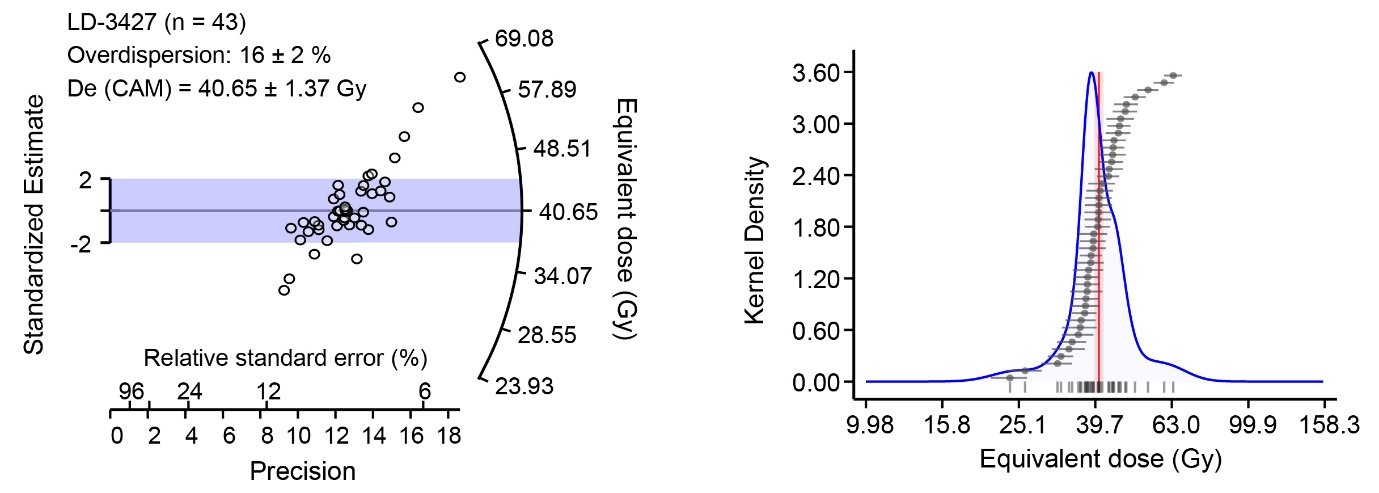


Figure S6. Galbraith Radial Plot and Kernel Density Plot of equivalent dose (De) distributions for sediment samples collected from the excavated trench at Chenga, Darjeeling plotted using LDAC program (Liang and Forman, 2019).


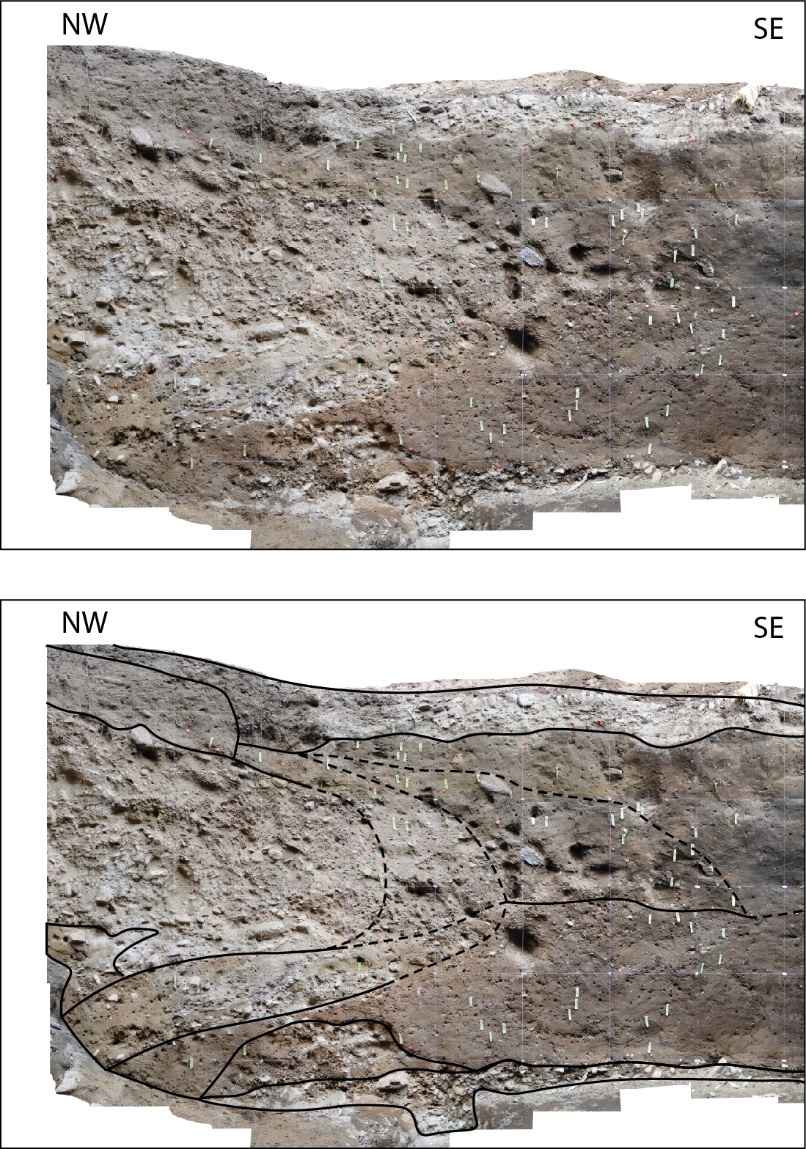
Figure S7. Close-up view of the main fault expression of trench exposure with and without different inferred units respectively.


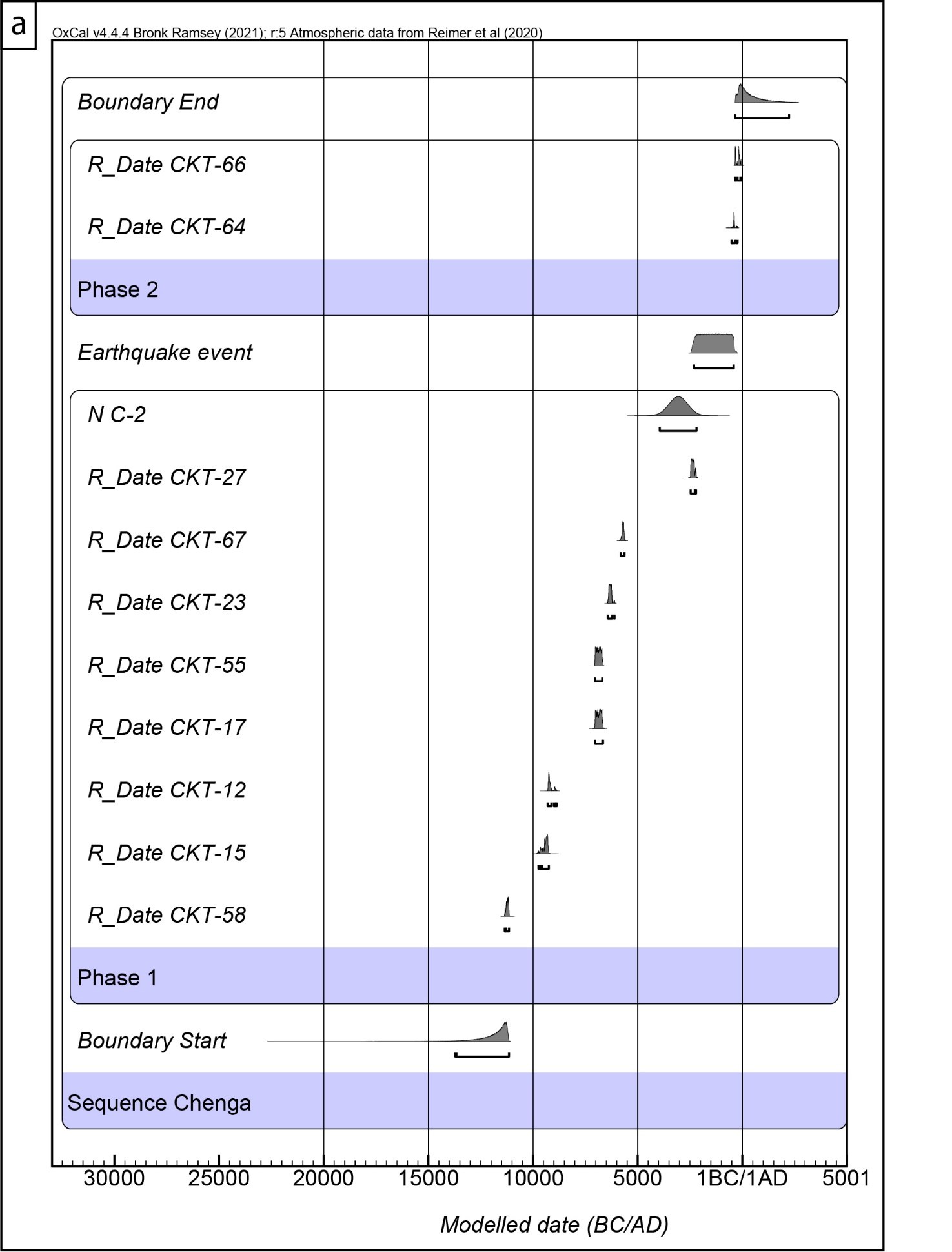

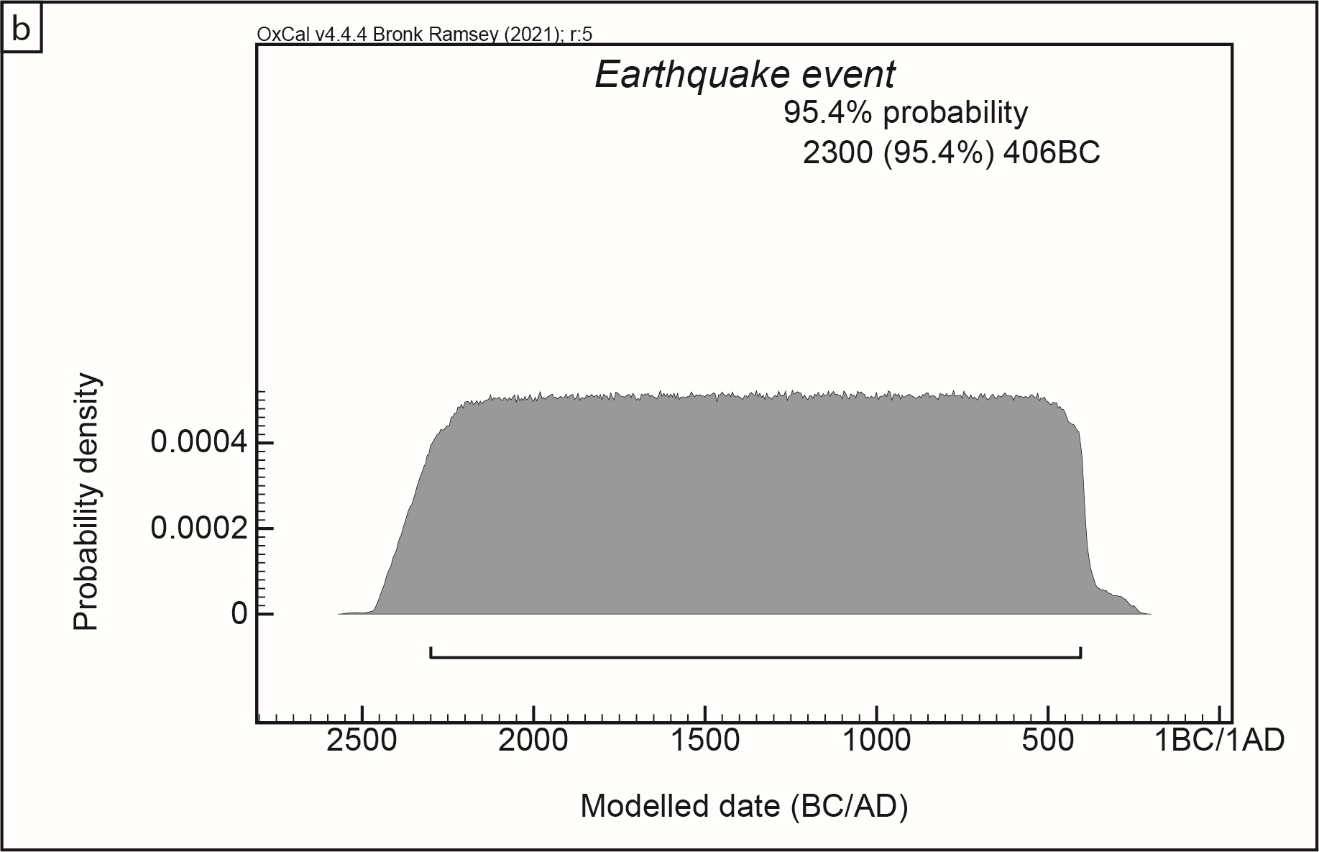
Figure S8. (A) OxCal model 4.4.4^4^ plot constraining surface rupturing earthquake event at Chenga village, Darjeeling. (B) Probability Density Function plot of the earthquake event at Chenga obtained using OxCal 4.4.4^4^.

| **Unit** | **Sample name^a^** | | **Lab. no.^b^** | | **Uncalibrated conventional radiocarbon age** | | **Calibrated ages (calendric, 2σ, 95.4%)^c^** | | **Probability distribution of 2σ ages** |
| --- | --- | --- | --- | --- | --- | --- | --- | --- | --- |
| **Excavated trench at the base of fault scarp at Chenga village** | | | | | | | | | |
| U7 | CKT-15 | | Poz-94980 | | 9900 ± 70 BP | | 9738-9247 BCE | | 9738-9723 BCE (0.9%)  9670-9571 BCE (10.9 %)  9561-9247 BCE (83.6%) |
| U7 | CKT-17 | | Poz-95030 | | 7940 ± 50 BP | | 7040-6657 BCE | | 7040-6686 BCE (94.1%)  6666-6657 BCE (1.3%) |
| U9 | CKT-23 | | Poz-94737 | | 7430 ± 50 BP | | 6422-6103 BCE | | 6422-6221 BCE (93.3%)  6128-6103 BCE (2.1%) |
| U9 | CKT-53 | | Poz-95032 | | 4175 ± 35 BP | | 2886-2631 BCE | | 2886-2662 BCE (91.3%)  2654-2631 BCE (4.1%) |
| U3 | CKT-58 | | Poz-95033 | | 11280 ± 60 BP | | 11350-11146 BCE | | 11350-11312 BCE (9.3%)  11307-11146 BCE (86.2%) |
| U10 | CKT-64 | | Poz-95034 | | 2325 ± 35 BP | | 514-229 BCE | | 514-500 BCE (1.2%)  487-354 BCE (83.4%)  284-229 BCE (10.9%) |
| U10 | CKT-66 | | Poz-95036 | | 2150 ± 30 BP | | 353-54 BCE | | 353-285 BCE (27.8%)  229-92 BCE (63.1%)  77-54 BCE (4.6%) |
| U4 | CKT-67 | | Poz-94738 | | 6820 ± 50 BP | | 5802-5626 BCE | | 5802-5626 BCE (95.4%) |
| U6 | CKT-68 | | Poz-95037 | | 12290 ± 60 BP | | 12866-12113 BCE | | 12866-12752 BCE (13.1%)  12565-12113 BCE (82.4%) |
| U7 | CKT-9 | | IUAC#17C984 | | 10485 ± 53 | | 10726-10154 BCE | | 10726-10480 BCE (67.7%)  10448-10371 BCE (8.6%)  10364-10299 BCE (9.1%)  10291-10221 BCE (9.0%)  10170-10154 BCE (1.0%) |
| U7 | CKT-12 | | IUAC#17C985 | | 9729 ± 52 | | 9300-8866 BCE | | 9300-9121 BCE (82.8%)  9001-8923 BCE (11.1%)  8893-8866 BCE (1.6%) |
| U9 | CKT-24 | | IUAC#17C986 | | 8060 ± 50 | | 7176-6775 BCE | | 7176-6811 BCE (94.2%)  6794-6775 BCE (1.3%) |
| U9 | CKT-27 | | IUAC#17C987 | | 3882 ± 43 | | 2469-2206 BCE | | 2469-2275 BCE (85.5%)  2256-2206 BCE (10.0%) |
| U10 | CKT-50 | | IUAC#17C988 | | 2775 ± 41 | | 1015-821 BCE | | 1015-821 BCE (95.4%) |
| U4 | CKT-55 | | IUAC#17C989 | | 7951 ± 49 | | 7041-6691 BCE | | 7041-6691 BCE (95.4%) |
| U7 | CKT-62 | | IUAC#17C990 | | 8404 ± 50 | | 7581-7347 BCE | | 7581-7445 BCE (68.4%)  7439-7347 BCE (27.1%) |
| **Excavated pit on the surface of T2 Terrace at Chenga village** | | | | | | | | |  |
| U2 | P-1 | | IUACD#17C1358 | | 4061± 40 | | 2851-2472 BCE | | 2851-2810 BCE (9.5%)  2747-2727 BCE (2.9%)  2698-2472 BCE (83.1%) |
| U3 | P-2 | | IUACD#17C1359 | | 82± 34 | | 1685-1929 CE* | | 1684-1734 CE (26.1%)  1803-1929 CE (69.4%) |
| U2 | P-3 | | IUACD#17C1360 | | 366± 34 | | 1451-1635 CE* | | 1451-1529 CE (48.2%)  1541-1635 CE (47.3%) |
| U2 | P-4 | | IUACD#17C1361 | | 3427± 69 | | 1901-1536 BCE | | 1901-1536 BCE (94.5%) |
| U3 | P-5 | | IUACD#17C1362 | | Sample could not be graphitized due to less carbon content | | | | |
| **Excavated pit on the surface of truncated QT1 terrace of Neora River^#^** | | | | | | | | | |
| Terrace | T2A | 133379 | | 3150±30 | | 1500-1314 BCE | | 1500-1383 BCE (87%)  1341-1314 BCE (8.5%) | |
| Terrace | T2E | 133380 | | 3115±30 | | 1447-1286 BCE | | 1447-1286 BCE (94.5%) | |

**Table- S1**: The calibrated charcoal samples were collected from the excavated trench at the base of the fault scarp and pit at the hanging wall of the faulted scarp, calibrated using OxCal 4.4.4^4^.

1. “CKT” series are charcoal samples collected from the excavated trench and the “P” series are charcoal samples collected from the excavated pit.
2. “Poz” series is the list of samples analysed at Poznań Radiocarbon Laboratory, Poznań, Poland and the “IUAC” series was analysed at IUAC, New Delhi, India.
3. Radiocarbon ages were calibrated using the Bayesian technique of OxCal 4.4.4^4^. All the reported radiocarbon dates are of 2σ (95.4% confidence level) calendar age ranges in BCE or CE.

* Unlike the rest of the charcoal samples collected from both trench and pit, these two samples show a very recent age comparatively. This can be justified as the shuffling of the charcoals due to anthropogenic activity causing recent charcoal to be buried at a depth of ~1m, as observed in the field.

# Charcoal samples from the pit excavated at the truncated QT1 terrace of Neora River^1^. The ages are recalibrated using OxCal 4.4.4^4^.

| **Unit** | **Field name** | **Lab No.** | **Depth (m)** | **Aliquots** | **Grain size (μm)** | **Equivalent dose (Gy)** | **OD (%)** | **U (ppm)^a^** | **Th**  **(ppm) ^a^** | **K (%)^a^** | **H2O (%)** | **Cosmic dose (mGy/yr)^b^** | **Dose Rate (mGy/yr) ^b^** | **Age BP (year) ^b^** | **Calibrated age ^c^** |
| --- | --- | --- | --- | --- | --- | --- | --- | --- | --- | --- | --- | --- | --- | --- | --- |
| **Excavated trench at the base of fault scarp at Chenga Village** | | | | | | | | | | | | | | | |
| U-8 | C-1 | LD-3427 | 3 | 43/43 | 75-125 | 40.65 ± 1.37 | 16 ± 2 | 2.6 ± 0.26 | 36 ± 3.6 | 1.76 ± 0.18 | 5 ±0.5 | 0.17 ± 0.02 | 4.87 ± 0.31 | 8375 ± 615 | 7586-5126 BCE |
| U-9 | C-2 | LD-3428 | 1.2 | 39/39 | 75-125 | 21.44 ± 1.45 | 30 ± 3 | 3.4 ± 0.34 | 23.5 ± 2.35 | 1.76 ± 0.18 | 5 ±0.5 | 0.18 ± 0.02 | 4.22 ± 0.25 | 5075 ± 445 | 3946-2166 BCE |
| **Excavated pit at the surface of T2 terrace at Lohargarh Forest** | | | | | | | | | | | | | | | |
| U-2 | LFO-1 | LD-3667 | 1.2 | 38/38 | 75-125 | 14.87 ± 0.69 | 20 ± 3 | 3.6 ± 1.2 | 20.3 ± 2.03 | 75-125 | 5 ± 0.5 | 0.18 ± 0.02 | 4.44 ± 0.27 | 3340 ± 260 | 1840-800 BCE |

**Table S2.** OSL data summary generated from Single Aliquot Regeneration method^6^ using RISO-TL/OSL system at Wadia Institute of Himalayan Geology (WIHG), Dehradun.

1. U, Th and K concentrations measured at XRF lab, WIHG, Dehradun. Uncertainty of the concentration is ~10%.
2. Luminescence dose and age calculated using excel based program (LDAC)^5^.
3. Ages were calibrated using the Bayesian technique of OxCal v4.4.4^4^.

References

1. Kumar, S., Wesnousky, S.G., Jayangondaperumal, R., Nakata, T., Kumahara, Y. and Singh, V. Paleoseismological evidence of surface faulting along the northeastern Himalayan front, India: Timing, size, and spatial extent of great earthquakes. Journal of Geophysical Research: Solid Earth, 115(B12), (2010).
2. Mukul, M. First-order kinematics of wedge-scale active Himalayan deformation: insights from Darjiling–Sikkim–Tibet (DaSiT) wedge. Journal of Asian Earth Sciences, 39(6), pp.645-657, (2010).
3. Mishra, R.L., Singh, I., Pandey, A., Rao, P.S., Sahoo, H.K. and Jayangondaperumal, R. Paleoseismic evidence of a giant medieval earthquake in the eastern Himalaya. Geophysical Research Letters, 43(11), pp.5707-5715, (2016).
4. Bronk Ramsey, C., 2009, Bayesian Analysis of Radiocarbon Dates: Radiocarbon, v. 51, p. 337–360, doi: 10.1017/S0033822200033865.
5. Liang, P. and Forman, S.L., 2019. LDAC: An Excel-based program for luminescence equivalent dose and burial age calculations. ancient TL, 37(2), pp.21-40.
6. Murray, A.S. and Wintle, A.G., 2000, Luminescence dating of quartz using an improved single-aliquot regenerative-dose protocol. Radiation measurements, 32(1), pp.57-73, <https://doi.org/10.1016/S1350-4487(99)00253-X>.
